# A computational method for identifying an optimal combination of existing drugs to repair the action potentials of SQT1 ventricular myocytes

**DOI:** 10.1371/journal.pcbi.1009233

**Published:** 2021-08-12

**Authors:** Karoline Horgmo Jæger, Andrew G. Edwards, Wayne R. Giles, Aslak Tveito

**Affiliations:** 1 Simula Research Laboratory, Oslo, Norway; 2 Department of Pharmacology, University of California, Davis, California United States of America; 3 Department of Physiology and Pharmacology, Faculty of Medicine, University of Calgary, Calgary, Canada; University of Michigan, UNITED STATES

## Abstract

Mutations are known to cause perturbations in essential functional features of integral membrane proteins, including ion channels. Even restricted or point mutations can result in substantially changed properties of ion currents. The additive effect of these alterations for a specific ion channel can result in significantly changed properties of the action potential (AP). Both AP shortening and AP prolongation can result from known mutations, and the consequences can be life-threatening. Here, we present a computational method for identifying new drugs utilizing combinations of existing drugs. Based on the knowledge of theoretical effects of existing drugs on individual ion currents, our aim is to compute optimal combinations that can ‘repair’ the mutant AP waveforms so that the baseline AP-properties are restored. More specifically, we compute optimal, combined, drug concentrations such that the waveforms of the transmembrane potential and the cytosolic calcium concentration of the mutant cardiomyocytes (CMs) becomes as similar as possible to their wild type counterparts after the drug has been applied. In order to demonstrate the utility of this method, we address the question of computing an optimal drug for the short QT syndrome type 1 (SQT1). For the SQT1 mutation N588K, there are available data sets that describe the effect of various drugs on the mutated K^+^ channel. These published findings are the basis for our computational analysis which can identify optimal compounds in the sense that the AP of the mutant CMs resembles essential biomarkers of the wild type CMs. Using recently developed insights regarding electrophysiological properties among myocytes from different species, we compute optimal drug combinations for hiPSC-CMs, rabbit ventricular CMs and adult human ventricular CMs with the SQT1 mutation. Since the ‘composition’ of ion channels that form the AP is different for the three types of myocytes under consideration, so is the composition of the optimal drug.

## Author summary

Poly-pharmacology (using multiple drugs to treat disease) has been proposed for improving cardiac anti-arrhythmic therapy for at least two decades. However, the specific arrhythmia contexts in which polytherapy is likely to be both safe and effective have remained elusive. Type 1 short QT syndrome (SQT1) is a rare form of cardiac arrhythmia that results from mutations to the human Ether-á-go-go Related Gene (hERG) potassium channel. Functionally, these mutations are remarkably consistent in that they permit the channel to open earlier during each heart beat. While hundreds of compounds are known to inhibit hERG channels, the specific effect of SQT1 mutations that allows for early channel opening also limits the ability of most of those compounds to correct SQT1 dysfunction. Here, we have applied a suite of ventricular cardiomyocyte computational models to ask whether polytherapy may offer a more effective therapeutic strategy in SQT1, and if so, what the likely characteristics of that strategy are. Our analyses suggest that simultaneous induction of late sodium current and partial hERG blockade offers a promising strategy. While no activators of late sodium current have been clinically approved, several experimental compounds are available and may provide a basis for interrogating this strategy. The method presented here can be used to compute optimal drug combinations provided that the effect of each drug on every relevant ion channel is known.

## Introduction

The action potentials of cardiomyocytes are governed by the dynamics of membrane proteins (ion channels) located at the myocyte membrane. Mutations affecting genes encoding one or more of the ion channels can significantly change the action potential (AP), see, e.g., [1–3], and some of these alterations can initiate dangerous arrhythmias [4–6]. The changes in the AP are often manifested in ECG recordings as prolonged or shortened QT-intervals, referred to as long-QT (LQT) or short-QT (SQT), respectively, see, e.g., [7–12]. In well diagnosed cases, treatment is available either in terms of anti-arrhythmic drugs [6, 13, 14] or in terms of an implantable cardioverter-defibrillator (ICD) [6, 10, 15], but at present both options have disadvantages: Many approved anti-arrhythmic drugs have serious side-effects and ICDs may fire inappropriatley and are difficult to apply for some patients [10, 14, 15]. Furthermore, some of the mutations that have been characterized are very rare [10, 16] which complicates both clinical identification and the development of new drugs.

Here, we propose a systematic strategy for identification of new drugs or combinations of drugs, based entirely on the selection of existing drugs. Our method is based on mathematical models of the AP coupled with models of how drugs influence the underlying ion currents. Mathematical models of the action potential of ventricular myocytes are well developed, see, e.g., [17–20]. These models have been extensively used to reveal the effect of changes to the ion channels, see, e.g., [21–23], and to attempt to understand the effects of various drugs, see, e.g., [24–27]. We have chosen to study models of the SQT1-syndrome where the KCHN2 (hERG) gene is altered, resulting in significant gain of function of the rapid delayed rectifier K^+^ current (*I*_Kr_). The increase of the *I*_Kr_-current leads to rapid repolarization and a shorter AP that in turn reduces the length of the QT-interval of the ECG. The reason for studying the SQT1-mutation is simply that there is data available describing the effect of this mutation on the *I*_Kr_-current *and* extensive data sets that characterize how a group of approved drugs affects the properties of that K^+^ current, see [28–30]. In future projects, pending similar data for other mutations, we can repeat the same steps to find theoretically optimal drugs.

Our method is based on the following assumptions:

1. The wild type (WT) and mutant (M) action potentials are well characterized by mathematical models.
2. A family of *K* existing drugs have been identified and characterized in terms of how each of these drugs affects the currents in the AP model.
3. Simple IC_50_/EC_50_-models (see below) can be applied to represent the effect of the drugs.
4. These models of the action of each drug are multiplicative (see below for an explicit definition) in the sense that the effect of several drugs can be multiplied in order to model their combined effect on a specific ion current.

Based on these assumptions, we can identify the best combination of the *K* different drugs, and then compare the new, theoretical, combined compound to the properties of the optimal versions of the existing drugs. For the selected ventricular myocyte target, we show, theoretically, that the combined compound clearly improves the mutant AP waveform more than any of the existing drugs utilized alone.

We apply this method to these AP models: human induced pluripotent stem cell-derived cardiomyocytes (hiPSC-CMs), rabbit ventricular cardiomyocytes, and adult human ventricular cardiomyocytes. The AP model applied here was developed for computational maturation [31], applied for identification of side effects of drugs in [32] and computational translation between species in [33]. In all cases we consider both wild type and mutant myocytes, and the challenge is to find a drug that, as applied to the mutant AP, becomes as similar to the wild type AP as possible as judged by a selected set of biomarkers (see the Methods section). We compute explicit concentrations of the different drugs in the combined compound also for rabbit or hiPSC-derived CMs in order to facilitate experimental testing using rabbit or hiPSC-derived cardiomyocytes.

## Methods

We consider mathematical models of the action potential written on the form,

$$\begin{array}{c} \frac{dv}{dt}=-\sum_{i}I_{i}, \end{array}$$ (1)

where *v* is the membrane potential (in mV), *t* denotes time (in ms) and *I*_*i*_ denotes membrane currents (in A/F). Individual ion channel currents can be written on the form

$$\begin{array}{c} I_{i}=\rho_{i}J_{i}, \end{array}$$ (2)

where

$$\begin{array}{c} \rho_{i}=\frac{N_{i}}{AC_{m}} \end{array}$$ (3)

and

$$\begin{array}{c} J_{i}=g_{0}^{i}o_{i}\left(v-E_{i}\right). \end{array}$$ (4)

Here, *A* is the area of the cell membrane (in *μ*m^2^), *C*_*m*_ is the specific capacitance of the cell membrane (in pF/*μ*m^2^), *N*_*i*_ is the number of channels of type *i*, $g_{0}^{i}$ is the conductance of a single open channel (in nS), *o*_*i*_ is the unitless open probability of the channel, and *E*_*i*_ is the electrochemical equilibrium potential of the channel (in mV).

Splitting *I*_*i*_ into *ρ*_*i*_ and *J*_*i*_ is convenient because it allows us to split the effect of mutation and maturation/translation. We assume that only *ρ*_*i*_ is changed during *maturation* from hiPSC-CMs to adult CMs, or by *translation* from animal CMs to human CMs. Likewise, only *J*_*i*_ is changed by the *mutation*. This also holds the other way around; *ρ*_*i*_ is independent of the mutation and *J*_*i*_ is independent of the maturation/translation.

### Modeling drug effects

We assume that we have a collection of *K* different drugs and we want to find an optimal combination of these drugs in order to ‘repair’ the effects of a mutation on the AP and the intracellular Ca^2+^ transient. In order to do this, we need a mathematical model of how each drug can affect the properties of a mutant ion channel. Since data on how drugs alter channel dynamics and conductance is limited, we base our analysis on a very simple model of drug effects (IC_50_/EC_50_). However, the same procedure is applicable if data is available to allow more elaborate, and accurate, representation of drug effects using Markov models.

We assume that both ion channel blockers (antagonists) and openers (agonists) will be encountered and therefore we need a formalism than can encompass both cases. To this end, we assume that the effect of a drug on a current *I* can be written in the form

$$\begin{array}{c} I\left(D\right)=(1+\frac{{\left(\varepsilon D\right)}^{H}}{{\left(\varepsilon D\right)}^{H}+1}E)I\left(0\right). \end{array}$$ (5)

Here, *D* denotes the concentration of the drug, *E* is the maximum effect of the drug, *H* is the Hill coefficient, and EC_50_ = 1/*ε* is the concentration that gives half maximum effect of the drug. The relative change of the current due to the drug is given by

$$\begin{array}{c} \eta \left(D\right)=\frac{I(D)-I(0)}{I(0)}=\frac{{\left(\varepsilon D\right)}^{H}}{{\left(\varepsilon D\right)}^{H}+1}E. \end{array}$$ (6)

We observe that *η*(0) = 0, *η*(1/*ε*) = *E*/2 and *η*(∞) = *E*. In order to use this model, we need to be able to estimate *ε*, *H* and *E* from data describing how the drug affects the properties of ion currents of the myocyte carrying the mutation. If the drug is a blocker, it is often convenient to use *E* = −1 and then (5) takes the usual form of the IC_50_ model;

$$\begin{array}{c} I\left(D\right)=\frac{1}{{\left(\varepsilon D\right)}^{H}+1}I\left(0\right). \end{array}$$ (7)

where 1/*ε* = IC_50_. Note that in estimating *E*, there is an obvious lower bound (*E* = −1), but there is no obvious upper limit.

#### Drug effects on the AP model

We assume that we have *K* different, existing, drugs and for each drug, *k*, we have determined $E_{i}^{k}$, $\varepsilon_{i}^{k}$ and $H_{i}^{k}$ for each current *i* that contribute to the AP, as discussed above. As a consequence, the model of the AP under the influence of a specific drug *k* is given by

$$\begin{array}{c} \frac{dv}{dt}=-\sum_{i}I_{i}\left(D\right)=-\sum_{i}(1+\frac{{\left(\varepsilon_{i}^{k}D\right)}^{H_{i}^{k}}}{{\left(\varepsilon_{i}^{k}D\right)}^{H_{i}^{k}}+1}E_{i}^{k})I_{i}\left(0\right). \end{array}$$ (8)

### Multiplicative effect of combined drugs

In principle, we can combine many drugs while searching for an optimal composition, but practical considerations suggest that only a few drugs (2 or 3) should be combined. By applying a combination of *K* drugs to the *i*-th current in the mutant model, we find that

$$\begin{array}{c} I_{i}\left(D\right)=\Pi_{k=1}^{K}(1+\frac{{\left(\varepsilon_{i}^{k}D_{k}\right)}^{H_{i}^{k}}}{{\left(\varepsilon_{i}^{k}D_{k}\right)}^{H_{i}^{k}}+1}E_{i}^{k})I_{i}\left(0\right). \end{array}$$ (9)

In order to simplify this notation, we let the properties of the *k*-th drug be denoted by $\Delta_{k}=\left\{E_{i}^{k},\varepsilon_{i}^{k},H_{i}^{k}\right\}$ where *i* runs over all the transmembrane currents. Furthermore, we let Δ denote the combination of the *K* drugs given by ${\left\{\Delta_{k}\right\}}_{k=1}^{K}$. The vector of doses is given by $D={\left\{D_{k}\right\}}_{k=1}^{K}$.

The AP model after the combination of drugs has been applied now takes the form,

$$\begin{array}{c} \frac{dv^{\Delta }}{dt}=-\sum_{i}F_{i}\left(D;\Delta \right)I_{i}\left(0\right), \end{array}$$ (10)

where

$$\begin{array}{c} F_{i}\left(D;\Delta \right)=\Pi_{k=1}^{K}(1+\frac{{\left(\varepsilon_{i}^{k}D_{k}\right)}^{H_{i}^{k}}}{{\left(\varepsilon_{i}^{k}D_{k}\right)}^{H_{i}^{k}}+1}E_{i}^{k}). \end{array}$$ (11)

The rationale and merits of assumption (9) are discussed below, and in the S1 Text.

### Identifying the optimal composition of drugs

As mentioned above, we wish to identify a set of optimal doses $D={\left\{D_{k}\right\}}_{k=1}^{K}$ for a set of *K* different drugs with known properties $\Delta_{k}=\left\{E_{i}^{k},\varepsilon_{i}^{k},H_{i}^{k}\right\}$ so that the AP and Ca^2+^ transient of the drug-treated mutant cardiomyocytes very closely approximate the AP and Ca^2+^ transient of wild type cardiomyocytes. To this end, we consider an action potential model where the effect of the mutation is represented such that a mutated and a wild type version of the model are defined and can be easily compared. Furthermore, we estimate the optimal doses *D* by minimizing a cost function measuring the difference between the model solutions.

#### Cost function definition

**Fig 1:**
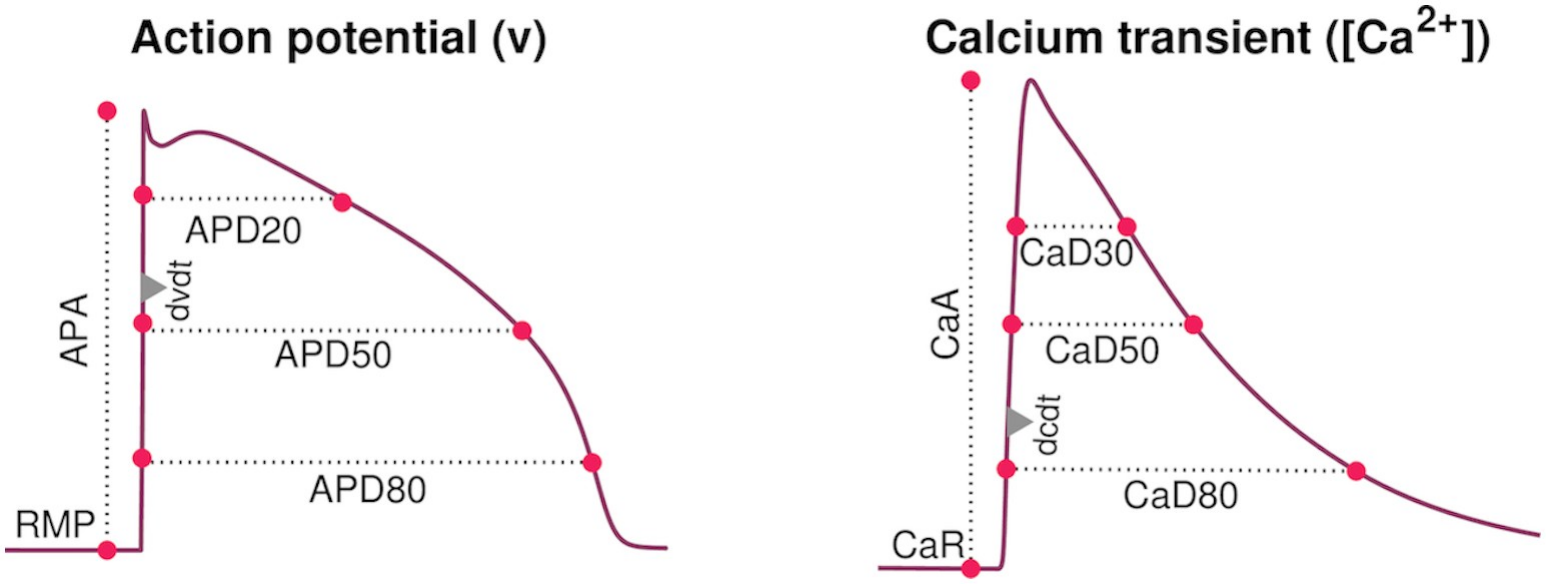
Illustration of the AP and Ca^2+^ transient biomarkers utilized in the cost function employed to identify optimal drug concentrations. From the AP, we consider the resting membrane potential (RMP), the AP amplitude (APA), the maximal upstroke velocity (dvdt) and the AP duration at different percentages of repolarization (APD10, APD50, …, APD90). From the cytosolic Ca^2+^ transient, we consider the resting Ca^2+^ concentration (CaR), the Ca^2+^ transient amplitude (CaA), the maximal upstroke velocity (dcdt) and the calcium transient durations CaD30, CaD50, and CaD80.

We utilize the cost function

$$\begin{array}{c} C\left(D\right)=\sum_{j}w_{j}\frac{|R_{j}^{M}\left(D\right)-R_{j}^{W}|}{|R_{j}^{W}|}, \end{array}$$ (12)

where $R_{j}^{W}$ represent different biomarkers for the wild type AP model, $R_{j}^{M}\left(D\right)$ represent the corresponding biomarkers for the mutant model with the drug doses *D* applied, and *w*_*j*_ are weights for the different biomarkers. More specifically, we consider the biomarkers RMP (resting membrane potential), APA (action potential amplitude), dvdt (maximal upstroke velocity of the action potential), APD10, APD20, …, APD90 (action potential duration at 10%, 20%, …, 90% repolarization), CaR (resting cytosolic Ca^2+^ concentration), CaA (cytosolic Ca^2+^ transient amplitude), dcdt (maximal upstroke velocity of the cytosolic Ca^2+^ transient), and CaD30, CaD50, and CaD80 (cytosolic Ca^2+^ transient duration at 30%, 50% and 80% of the maximum amplitude). The definition of these biomarkers are illustrated in Fig 1. In the cost function (12), we use the weight *w*_*j*_ = 1 for all terms except that the weights for APD80, APD90 and dvdt are set to 5.

#### Minimization procedure

The problem of identifying the minimium of (12) clearly grows in complexity as the number of drugs increases. Here, we use an approach that gradually increases the number of drugs and thus assures that we have a reasonably good initial guess for every minimization problem.

In the case of one drug, finding the optimal dose of that drug is a straightforward minimization of the cost function (12) with only one free parameter (the dose of the single drug). Suppose you have found the optimal combination of *n* drugs (where *n* < *K*). Next, you want to see if you can use one of the remaining *K* − *n* drugs to improve the approximation of the wild type case. The problem is then to solve *K* − *n* minimization problems with *n* + 1 parameters. The minimization is now started using the best solution for *n* drugs as an initial guess, and for the one additional drug, we set the initial dose to be zero. The optimal solution of this *n* + 1 dimensional problem is solved using the continuation method of [12, 32]. This is repeated for the *K* − *n* remaining drugs, and the best solution is stored as the optimal drug for the case of combining *n* + 1 drugs. The process is repeated until *n* = *K*. Further technical specifications of the applied minimization procedure are provided in S1 Text.

### The channel block/agonist model is unchanged during maturation or species translation

As in [33], we assume that the properties $\left\{E_{i}^{k},\varepsilon_{i}^{k},H_{i}^{k}\right\}$ of a drug *k* on a specific ion channel *i* is the same for animal and human cells. The methods described in this report could therefore, in principle, be applied to find optimal drug compounds for adult human CMs based on data from hiPSC-CMs or an animal (e.g., rabbit). We will explain this in some detail for the case of using data from hiPSC-CMs to define models of how the drug affects adult CMs. Recall that the currents in the model are written on the form *I* = *ρJ* where the factor *ρ* changes during maturation, but is unaltered by the mutations; and, vice versa. That is, the function *J* is unchanged by maturation but is altered by the mutation. To be explicit, for a given ion current we have

$$\begin{array}{cc} I^{IM,M} & =\rho^{IM}J^{M}, \end{array}$$ (13)

$$\begin{array}{cc} I^{A,M} & =\rho^{A}J^{M}, \end{array}$$ (14)

where *IM*, *A*, and *M* is for immature, adult and mutant, respectively. Recall that for an ion channel, *J* = *g*_0_*o*(*v* − *E*), and, under the influence of the drug, we have *J* = *J*(*D*) = *F*(*D*)*g*_0_*o*(*v* − *E*). Since *J* is the same for *IM* and *A*, the effect of the drug is also same, and thus we have

$$\begin{array}{cc} I^{IM,M}\left(D\right) & =\rho^{IM}J^{M}\left(D\right), \end{array}$$ (15)

$$\begin{array}{cc} I^{A,M}\left(D\right) & =\rho^{A}J^{M}\left(D\right). \end{array}$$ (16)

Therefore, if we estimate *ε*, *H* and *E* in the model (5) from measurements of hiPSC-CMs (e.g., using the computational inversion procedure of [32]), these values are also the correct values in the adult case. Exactly the same argument can be used to translate from rabbit data to *ε*, *H* and *E* values for adult human CMs. The reason why this is possible rests on the assumption that the effect of the drug on a specific ion channel is the same regardless of whether it is expressed in hiPSC-derived myocytes, rabbit myocytes or myocytes from adult humans.

### Modeling the SQT1 mutation

The specific action potential model used in our computations is an updated version of the base model initially published in [32]. More specifically, we use the updated base model formulation described [12]. In that model, the *I*_Kr_ current is fitted to data from measurements of wild type *I*_Kr_ currents and *I*_Kr_ currents for the SQT1 mutation N588K from [34]. In particular, the voltage dependence of the steady state value of the inactivation gate, *x*_Kr2_, of the *I*_Kr_ current is shifted towards more positive potentials:

$$\begin{array}{ccc} x_{\text{Kr}2,\infty } & =\frac{1}{1+e^{(v+70)/20.9}}, & \left(\text{for}\;\text{WT}\right) \end{array}$$ (17)

$$\begin{array}{ccc} x_{\text{Kr}2,\infty } & =\frac{1}{1+e^{\left(v+70-62)/(20.9\cdot 1.85\right)}}, & (\text{for}\;\text{N588K}). \end{array}$$ (18)

In Fig 5 of [12] the *I*_Kr_ model is compared to measurements from [34]. Furthermore, the hiPSC-CM version of the model has been fitted to data of wild type and SQT1 hiPSC-CMs from [28], and the adult human ventricular CM version of the model has been validated using adult human ECG measurements from [35] (see [12]). For the computations in the present study, we also consider a rabbit version of the AP model. The rabbit parameterization is based on the rabbit models from [33, 36] and is fitted to published SQT1 and wild type APD90 values for rabbit from [37]. The parameters of the rabbit version of the model are specified in S1 Text, and the remaining parameter values of the base model are found in [12].

### EMI model simulations of a strand of cells

In order to estimate changes in the conduction velocity (CV) of cardiac tissue and the QT interval of the ECG caused by the short QT mutation and/or by the application of drugs, we carry out spatial simulations with an *in silico* strand of connected ventricular myocytes using the EMI model. This approach represents the extracellular space (E), the cell membrane (M) and the intracellular space (I), see, e.g., [38–40]. The EMI model equations are solved using an MFEM [41, 42] finite element implementation of the splitting algorithm introduced in [43, 44]. Technical specifications of the domain geometry and the EMI model solver are provided in the S1 Text.

### Drug characteristics

In this study, we attempt to identify optimal combinations of drugs for repairing the effect of the SQT1 mutation, N588K, which alters the function of the potassium current *I*_Kr_. Specifically, this mutation markedly increases the size of the *I*_Kr_ current, leading to a shortening of the AP. In order to ‘repair’ this effect, we have evaluated a number of *I*_Kr_ blockers, attempting to reduce the *I*_Kr_ current. In addition, we consider two drugs that increase the *I*_CaL_ or *I*_NaL_ currents, as alternative approaches for lengthing the action potential duration in the ventricular myocytes carrying this mutation. The properties of the considered drugs are listed in Table 1. Here, the properties of the drugs quinidine, ivabradine, ajmaline and mexiletine are taken from [12], where they were estimated based on measurements of SQT1 hiPSC-CMs from [28, 29]. Furthermore, the effect of the drugs disopyramide, propafenone and amiodarone on SQT1 *I*_Kr_ currents are taken from [30]. The EC_50_-values are taken directly from the paper and the Hill coefficients are estimated from fitting the model (5) to the dose-dependent block reported in Figs 4 and 5 of [30]. Data describing the effect of these drugs on *I*_CaL_, *I*_Na_ and *I*_NaL_ are taken from the comprehensive drug studies [45, 46]. Finally, parameters describing the properties of the *I*_CaL_ and *I*_NaL_ agonists BAY K 8644 and veratridine are relatively rough estimates based on data presented in [47–49].

**Table 1:** Drug characteristics.

Drug	SQT1 *I*_Kr_				*I*_CaL_				*I*_Na_				*I*_NaL_				*I*_f_			
	EC_50_	*H*	*E*	Ref.	EC_50_	*H*	*E*	Ref.	EC_50_	*H*	*E*	Ref.	EC_50_	*H*	*E*	Ref.	EC_50_	*H*	*E*	Ref.
Quinidine	8.14 *μ*M	1	−1	[12, 28]	153 *μ*M	1	−1	[12, 28]	77.7 *μ*M	1	−1	[12, 28]								
Ivabradine	12.6 *μ*M	1	−1	[12, 29]					86.3 *μ*M	1	−1	[12, 29]					42 *μ*M	1	−1	[12, 29]
Ajmaline	69.5 *μ*M	1	−1	[12, 29]	46.6 *μ*M	1	−1	[12, 29]	435 *μ*M	1	−1	[12, 29]								
Mexiletine	281 *μ*M	1	−1	[12, 29]	963 *μ*M	1	−1	[12, 29]	201 *μ*M	1	−1	[12, 29]								
BAY K 8644					0.05 *μ*M	1.7	1.8	[47, 48]												
Veratridine													0.426 *μ*M	2	1.8	[49]				
Disopyramide	15.77 *μ*M	0.6	−1	[30]	1036.7 *μ*M	1	−0.779	[45]	168.4 *μ*M	1.09	−0.311	[45]								
Propafenone	0.95 *μ*M	0.7	−1	[30]	1.55 *μ*M	0.9	−1	[46]	3.886 *μ*M	0.9	−1	[46]	4.036 *μ*M	0.9	−1	[46]				
Amiodarone	0.318 *μ*M	0.5	−1	[30]	1.28 *μ*M	0.6	−1	[46]	4.58 *μ*M	0.7	−1	[46]	9.42 *μ*M	0.4	−1	[46]				

Characteristics of selected drugs obtained from literature in the form of EC_50_-values (1/*ε*), Hill coefficients (*H*) and maximum effects (*E*) for the modified *I*_Kr_ current affected by the SQT1 mutation and for the wild type *I*_CaL_, *I*_Na_, *I*_NaL_ and *I*_f_ currents, see (5).

## Results

The main result of this study is to demonstrate that mathematical models of the ventricular myocyte action potential and calcium handling, coupled with models of how drugs interact with ion channels, can be used to find optimal drug combinations for anti-arrhythmic therapy. We show, theoretically, that the effects of the SQT1 mutation N588K can be repaired by searching for and then applying an optimal combination of existing drugs.

### SQT1 mutation in hiPSC-CMs, rabbit CMs and adult human CMs

**Fig 2:**
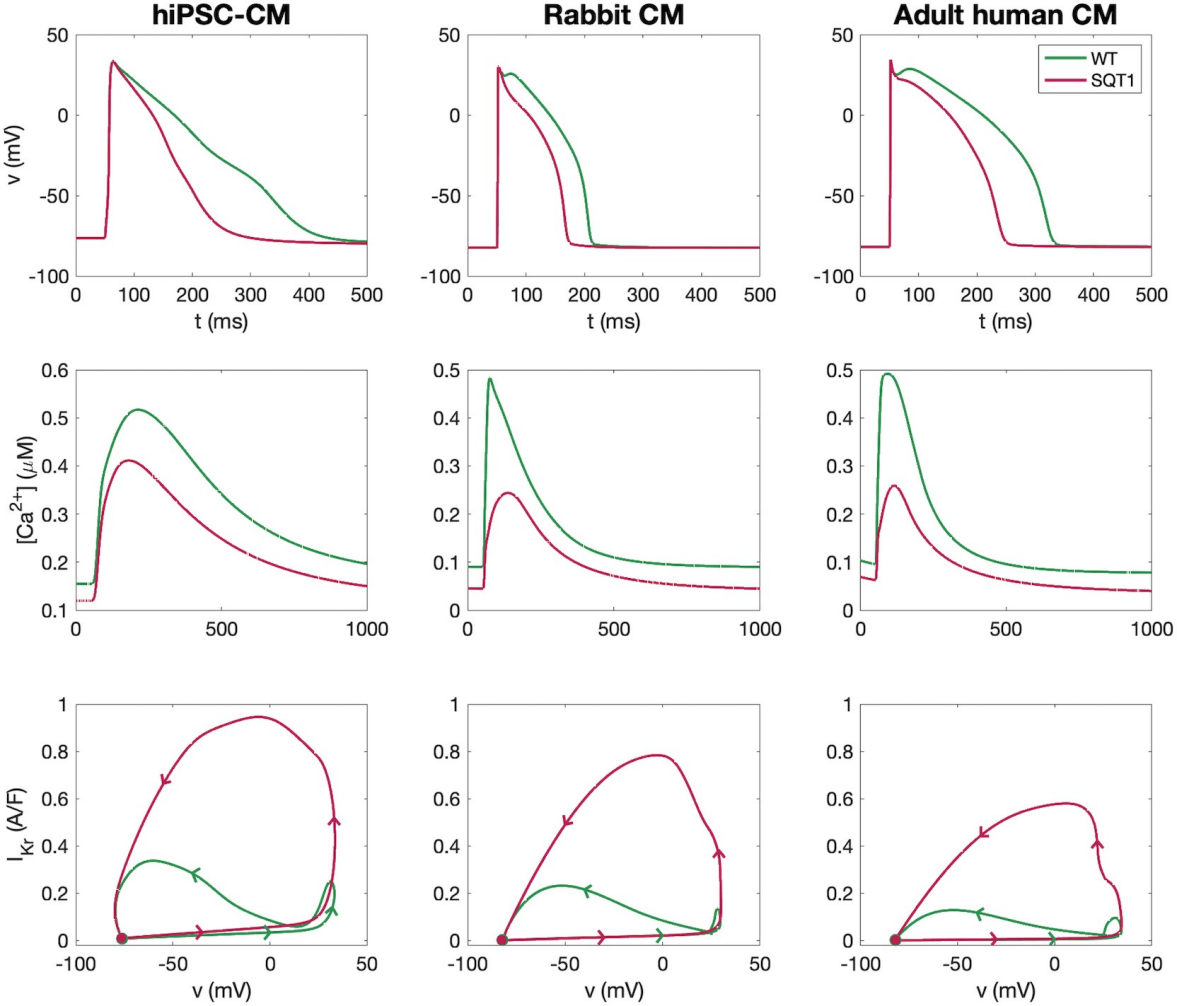
Action potentials, Ca^2+^ transients and *I*_Kr_ currents generated using our models for wild type and SQT1 hiPSC-CMs, rabbit ventricular CMs and adult human ventricular CMs. Each panel in the upper row shows the action potentials in the wild type and SQT1 cases, and the middle row shows the Ca^2+^ transients. In the lower row, the *I*_Kr_ current from each action potential simulation is plotted as a function of the membrane potential during the entire AP waveform. Here, the filled circles mark the solution at *t* = 0 and the arrows indicate the direction with time. Data used in this figure can be found in S1 Data.

In Fig 2, we show the action potentials (APs), Ca^2+^ transients and *I*_Kr_ currents generated by the wild type and SQT1 versions of the mathematical models for hiPSC-CMs, rabbit ventricular CMs and human adult CMs. The upper panel shows that for all these models, the AP is significantly shorter in the SQT1 case than in the wild type case. This reduced action potential duration is consistent with the short duration of the QT interval of the ECG that is characteristic of short QT syndrome. In addition, the middle panel of Fig 2 shows that the amplitude of the Ca^2+^ transient is reduced for the SQT1 situation compared to the wild type case, especially for rabbit and adult human CMs. Such reduced Ca^2+^ transient amplitudes have been observed in earlier computational studies of the N588K SQT1 mutation [50, 51], and this effect is in agreement with speckle-tracking echocardiography and Doppler imaging that have shown decreased left ventricular contraction in patients with SQT syndrome [52, 53]. The main goal of this study is to find combinations of drugs that alter the currents in the SQT1 case so that the AP and Ca^2+^ transient becomes very similar to the wild type AP and Ca^2+^ transient.

The SQT1 mutation affects the function of the *I*_Kr_ current; the only difference between the wild type and SQT1 versions of the AP models is a difference in the formulation of the *I*_Kr_ current, see [12]. In the lower panel of Fig 2, we compare the wild type and SQT1 *I*_Kr_ currents by plotting these currents from each of the two action potential simulations as functions of the membrane potential. Note that the *I*_Kr_ current is significantly larger in the SQT1 case than in the wild type case. We also observe that the voltage dependence is different in the SQT1 case compared to the wild type case. This indicates that drug effects implemented only in terms of altered maximum conductance, resulting from a pore block approach, for the *I*_Kr_ current of the form (5) will likely not completely eliminate the effect of the SQT1 mutation on the *I*_Kr_ current. Accordingly, instead of trying to repair the mutated *I*_Kr_ current directly, we instead attempt to ‘repair’ the effect of the mutation on the full action potential by minimizing the cost function (12) as detailed in the remainder of this Results section.

### Optimal combinations of two drugs

**Fig 3:**
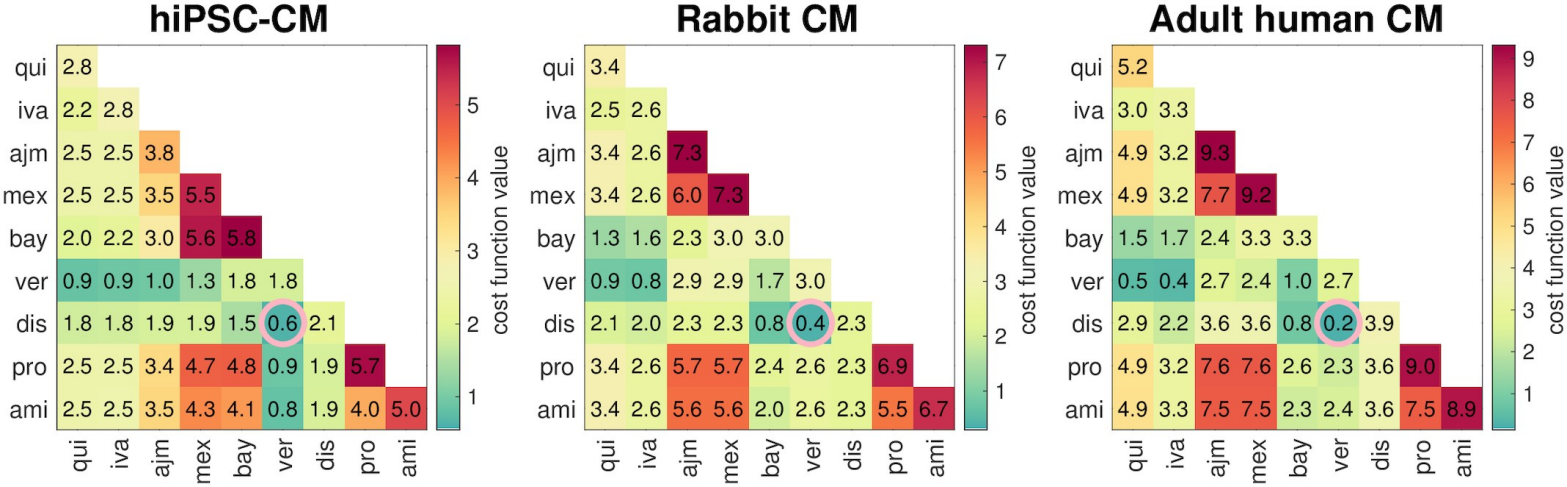
Optimal cost function values obtained by applying our computational procedure to combinations of two drugs, selected for their potential to repair the SQT1 mutation in hiPSC-CMs, rabbit ventricular CMs and adult human ventricular CMs. The numbers in the upper left to lower right diagonal report the cost function values found in searches for the optimal dose of a single drug. In addition, the pink circles indicate the lowest cost function value obtained in each case.

We first applied the computational procedure to search for optimal combinations of two drugs that may be capable of repairing the AP and Ca^2+^ transient of SQT1 CMs. Fig 3 illustrates our findings presented in terms of the minimum cost function value (12) for our procedure applied to each possible combination of two drugs from the list in Table 1. In addition, the numbers in the upper left to lower right diagonals report the optimal cost function values found in searches for the optimal dose of each single drug. Note that some of the combinations of drugs appear to result in relatively low cost function values, and that the optimal combinations of two drugs appear to result in considerably lower cost function values than the optimal doses of any single drug. In particular, the combination of veratridine and disopyramide, indicated by pink circles in Fig 3, appears to be the optimal combination of two drugs for both hiPSC-CMs, rabbit ventricular CMs and adult human ventricular CMs. Neither of these drugs, individually, appear to be able to completely repair the effect of the mutation.

**Fig 4:**
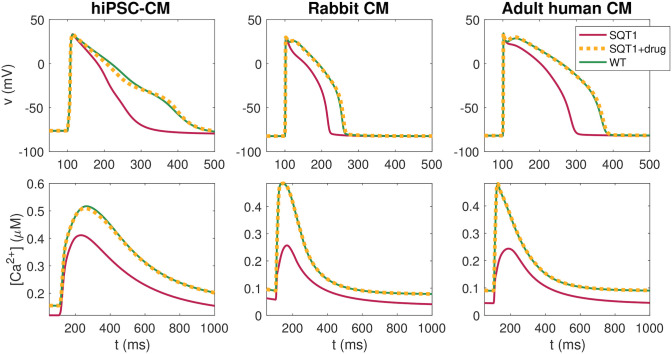
AP and Ca^2+^ transient for hiPSC-CMs, rabbit ventricular CMs and adult human ventricular CMs in the wild type case (solid green), in the SQT1 case (solid red), and in the SQT1 case with the optimal combination of two drugs from Fig 3 applied (dotted yellow). Data used in this figure can be found in S1 Data.

**Fig 5:**
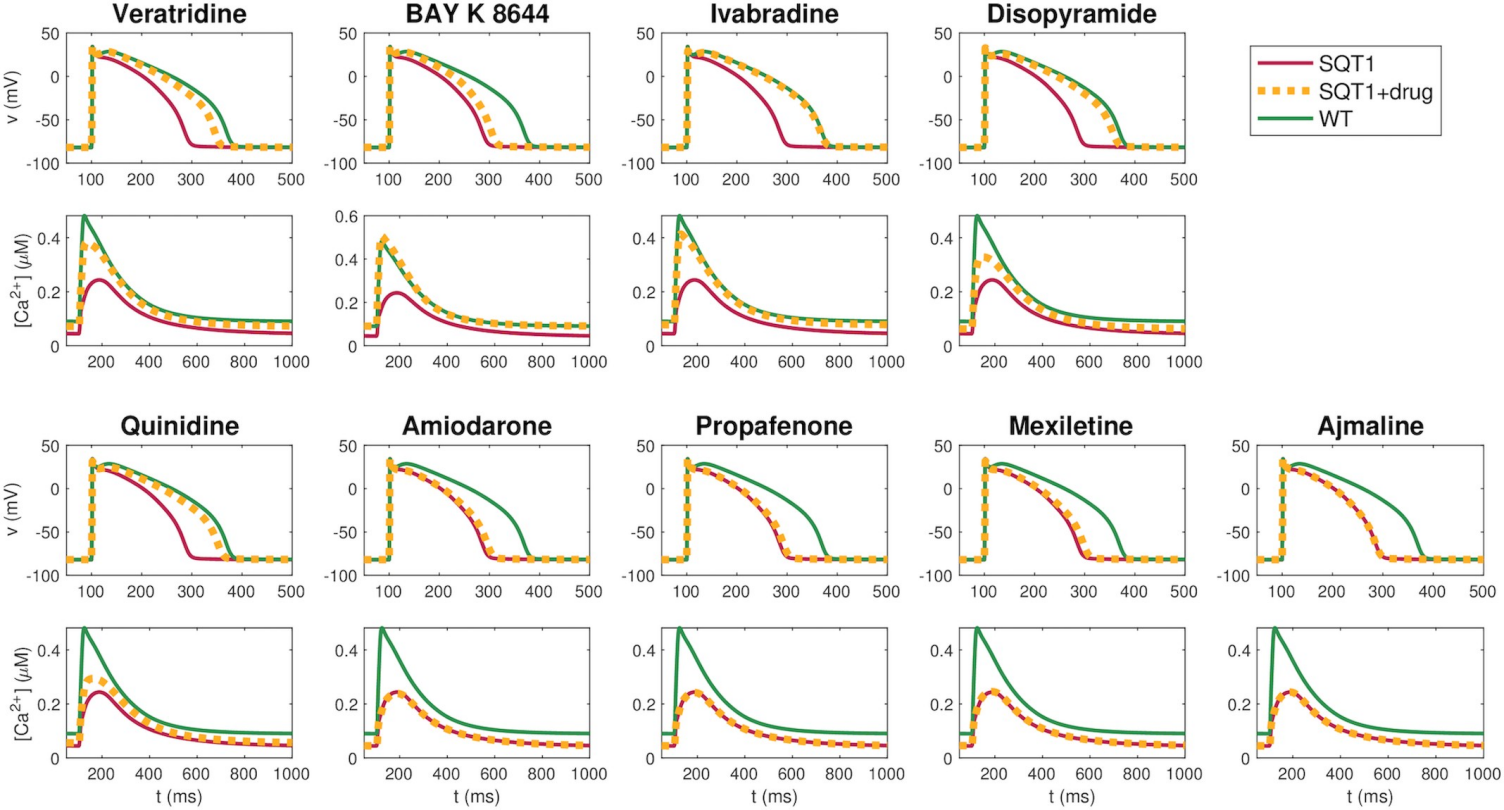
AP and Ca^2+^ transient for adult human ventricular myocytes in the wild type case, in the SQT1 case, and in the SQT1 case with the optimal dose of each of the drugs of Table 1 applied. The selected drugs are ordered from the smallest to the highest obtained cost function values. The applied doses are specified in Table 3. Data used in this figure can be found in S1 Data.

Fig 4 shows the AP and Ca^2+^ transient of the SQT1 models under the influence of the optimal dose combination of these two drugs. We consider the hiPSC-CM case, the rabbit ventricular CM case and the adult human ventricular CM case, and compare AP and Ca^2+^ transients for wild type, SQT1 and SQT1 with the drugs applied. We observe that the optimal combination of two drugs appears to repair both the SQT1 AP and Ca^2+^ transient almost fully; that is, the solutions of the SQT1 models with the optimal drug combination applied seem to be very similar to the wild type solutions. For comparison, Fig 5 shows similar plots for the optimal dose of each of the individual drugs in the adult human ventricular myocyte case. We observe that some of the drugs appear to repair the SQT1 mutation quite well, but not as well as the optimal combination of two drugs. Similar plots are provided for hiPSC-CMs and rabbit CMs in the S1 Text.

The data in Table 2 provides further basis for evaluating the efficacy of the selected two drug combination. Biomarkers computed for the wild type and SQT1 adult human ventricular CM cases are listed, as well as for the SQT1 case with the optimal combination of two drugs and for the optimal dose of each single drug applied. Note that the combination drug approach repairs all the considered biomarkers in the SQT1 phenotype from deviating up to 35% from the wild type case, to only deviating up to 3% from the wild type case. In addition, we observe that for the optimal dose of ivabradine, which seemed to almost completely repair the AP and Ca^2+^ transient waveforms of the SQT1 CMs in Fig 5, the maximal upstroke velocity and the conduction velocity differ considerably from the wild type case, explaining the relatively high cost function value obtained for this drug. Similar tables for the hiPSC-CM and rabbit CM cases are provided in the S1 Text.

**Table 2:** Biomarkers and cost function values.

	Cost function	APD50		APD90		dvdt_max_		CV		QT	
		ms	% from WT	ms	% from WT	mV/ms	% from WT	cm/s	% from WT	ms	% from WT
WT (no drug)	0	223		272		215		54		283	
SQT1 (no drug)	9.3	146	−35%	189	−31%	216	+1%	54	+0%	203	−28%
Combination drug	0.2	224	+1%	276	+1%	214	−0%	53	−1%	274	−3%
Veratridine	2.7	200	−10%	248	−9%	215	+0%	53	−1%	244	−14%
BAY K 8644	3.3	167	−25%	207	−24%	214	−0%	53	−0%	208	−27%
Ivabradine	3.3	223	−0%	270	−1%	133	−38%	44	−18%	278	−2%
Disopyramide	3.9	209	−7%	259	−5%	191	−11%	51	−5%	264	−7%
Quinidine	5.2	206	−8%	255	−6%	167	−22%	48	−10%	261	−8%
Amiodarone	8.9	154	−31%	199	−27%	211	−2%	53	−1%	214	−25%
Propafenone	9.0	151	−32%	196	−28%	212	−1%	53	−1%	203	−28%
Mexiletine	9.2	157	−30%	201	−26%	172	−20%	49	−9%	215	−24%
Ajmaline	9.3	146	−35%	189	−31%	216	+1%	54	+0%	203	−28%

Cost function and biomarker values of the adult human ventricular CM models for wild type and SQT1 with no drugs present, as well as for the SQT1 model with the optimal combination of two drugs or the optimal dose of the individual drugs applied. The cost function value (see (12)), the action potential durations (APD50 and APD90), the maximal upstroke velocity of the action potential (dvdt_max_,) the conduction velocity (CV), and the QT interval are listed. In the SQT1 cases, we also report the percent difference from the wild type case.

The optimal doses determined for each drug and for the optimal combination of two drugs in the adult human case are given in Table 3. This table also reports the associated block or increase as a percentage for the individual currents. In addition, we provide the effect of the optimal doses in the form of the percentage of the maximum effect (*E*, see (5)) of the drug, for the current most prominently affected by the drug. Similar tables are given in the S1 Text for the rabbit and hiPSC-CM cases.

**Table 3:** Optimal doses and effect on the ion currents.

Drug	Optimal dose	(max % of *E*)		% change of currents				
				*I*_Kr_	*I*_CaL_	*I*_Na_	*I*_NaL_	*I*_f_
Combination of two drugs	1.86 *μ*M 3.03 *μ*M	(95%) (27%)	veratridine disopyramide	−27.1%	−0.2%	−0.4%	+171.0%	+0.0%
Veratridine	1.86 *μ*M	(95%)		+0.0%	+0.0%	+0.0%	+171.0%	+0.0%
BAY K 8644	0.0475 *μ*M	(48%)		+0.0%	+86.2%	+0.0%	+0.0%	+0.0%
Ivabradine	67.7 *μ*M	(84%)		−84.3%	+0.0%	−44.0%	+0.0%	−61.7%
Disopyramide	142 *μ*M	(79%)		−78.9%	−9.4%	−14.1%	+0.0%	+0.0%
Quinidine	29.3 *μ*M	(78%)		−78.2%	−16.1%	−27.4%	+0.0%	+0.0%
Amiodarone	0.0338 *μ*M	(25%)		−24.6%	−10.2%	−3.1%	−9.5%	+0.0%
Propafenone	0.0752 *μ*M	(14%)		−14.5%	−6.2%	−2.8%	−2.7%	+0.0%
Mexiletine	65.3 *μ*M	(25%)		−18.9%	−6.3%	−24.5%	+0.0%	+0.0%
Ajmaline	0.00204 *μ*M	(0.0044%)		−0.0%	−0.0%	−0.0%	+0.0%	+0.0%

Optimal doses of a single drug, or a combination of two drugs that can repair the SQT1 mutation in adult human ventricular CMs. In addition, we report the effect of the optimal dose of each drug in the form of the percentage of the maximum effect of the drug on the current that is most strongly affected by the drug (max % of *E*). The percent change of each of the currents resulting from the optimal doses is also presented.

### Optimal combinations of drugs, when emphasizing relatively low drug doses

**Fig 6:**
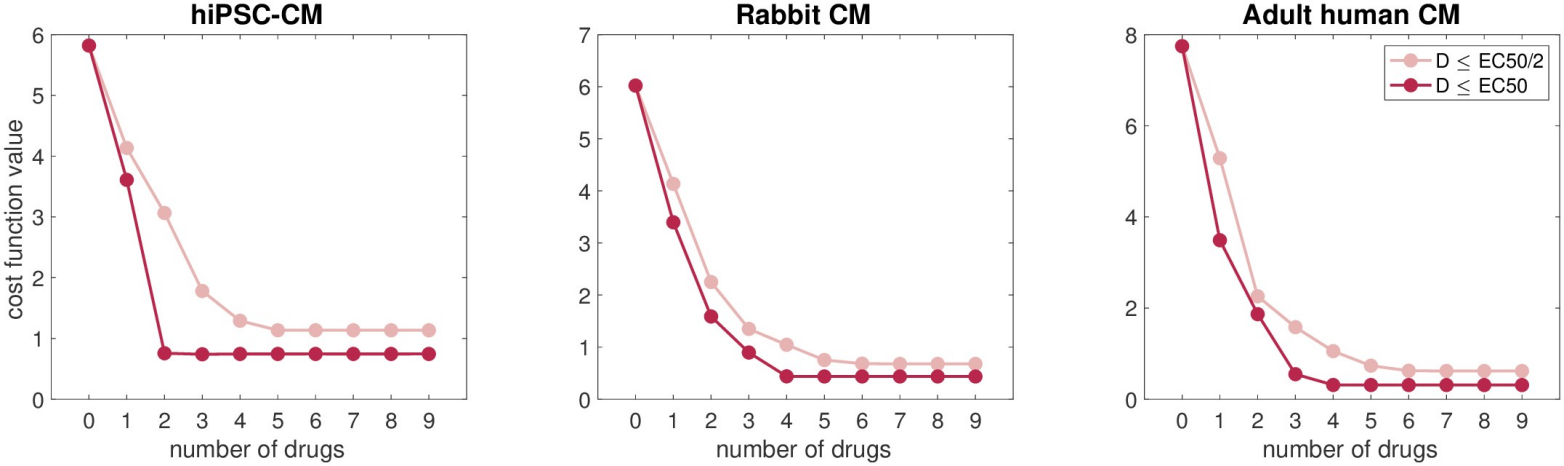
Optimal cost function values obtained when our computational procedure is applied to combinations of an increasing number of drugs applied simultaneously with the goal of repairing the SQT1 mutation in hiPSC-CMs, rabbit ventricular CMs and adult human ventricular CMs. These computations were done applying the restrictions *D* ≤ min(EC_50_)/2 (pink) and *D* ≤ min(EC_50_) (red) for the drug doses. Data used in this figure can be found in S1 Data.

Based on the optimal doses found for each single drug, and also for a combination of two drugs in Table 3, we recognize that the identified doses are quite high. For instance, the optimal dose of veratridine (1.86 *μ*M) is more than four times higher than the EC_50_-value of veratridine. This results in an enhancement of the *I*_NaL_ current that is 95% of the maximum effect of veratridine. In fact, *I*_NaL_ is increased by a factor of 2.8 (see Table 1). This is the maximal dose allowed in these applications of the computational procedure (see the S1 Text). In order to avoid potential side effects of high drug doses, it is generally beneficial to avoid such large doses. Therefore, we also wish to apply the computational procedure to find optimal drug combinations with lower drug doses. In Fig 6, we report the optimal cost function values found in the search for optimal drug combinations for an increasing number of drugs, combined with a strict limit on the maximal allowed drug doses. More specifically, we consider the restrictions *D* ≤ min(EC_50_)/2 and *D* ≤ min(EC_50_). We observe that for the restriction *D* ≤ min(EC_50_)/2, the cost function value is drastically decreased when only one or two drugs are applied, and then gradually decreased until 5–6 drugs are included. Furthermore, for the less strict condition *D* ≤ min(EC_50_), fewer drugs are needed to reduce the cost function value. In the hiPSC-CM case, it seems like 2 drugs are sufficient to achieve an optimal solution. For the rabbit and adult human cases, 4 drugs seem to provide an effective ‘repair’ of the AP and Ca^2+^ waveforms.

**Fig 7:**
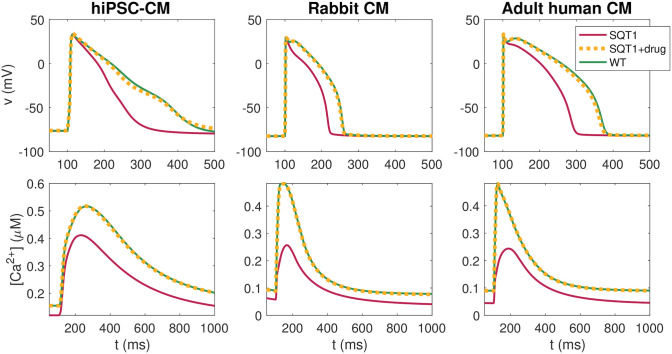
AP and Ca^2+^ transient waveforms for hiPSC-CMs, rabbit ventricular CMs and adult human ventricular CMs in the wild type case (solid green), in the SQT1 case (solid red), and in the SQT1 case with the optimal combination of five drugs with the restriction *D* ≤ min(EC_50_)/2 from Fig 6 applied (dotted yellow). Data used in this figure can be found in S1 Data.

The AP and Ca^2+^ transient for the optimal combinations of 5 drugs with the restriction *D* ≤ min(EC_50_)/2 are plotted in Fig 7. In addition, biomarkers of the solutions and the optimal drug doses are summarized in Tables 4 and 5 for the adult human case and in the S1 Text for the hiPSC-CM and rabbit cases. We observe that the combination of 5 drugs with the restriction *D* ≤ min(EC_50_)/2 on the doses seem to be able to repair the AP and Ca^2+^ transient of the SQT1 CMs quite well.

**Table 4:** Biomarkers for WT, SQT1 and SQT1 with drug.

	*C*	APD50		APD90		dvdt_max_		CV		QT	
		ms	% from WT	ms	% from WT	mV/ms	% from WT	cm/s	% from WT	ms	% from WT
WT (no drug)	0	223		272		215		54		283	
SQT1 (no drug)	9.3	146	−35%	189	−31%	216	+1%	54	+0%	203	−28%
Combination drug	0.7	217	−3%	267	−2%	197	−8%	52	−4%	278	−2%

Cost function and biomarker values for the SQT1 human ventricular CM model based on an optimal combination of five drugs with the restriction *D* ≤ min(EC_50_)/2 applied. The table follows the structure of Table 2.

**Table 5:** Optimal doses of five drug combinations.

Drug	Optimal dose	(max % of *E*)		% change of currents				
				*I*_Kr_	*I*_CaL_	*I*_Na_	*I*_NaL_	*I*_f_
Combination of five drugs	7.77 *μ*M	(40%)	disopyramide	−69.4%	+5.7%	−10.2%	+35.6%	−9.3%
	3.84 *μ*M	(32%)	quinidine					
	4.32 *μ*M	(26%)	ivabradine					
	0.211 *μ*M	(20%)	veratridine					
	0.00883 *μ*M	(5%)	BAY K 8644					

Optimal doses of a combination of five drugs with the restriction *D* ≤ min(EC_50_)/2 found for repairing the SQT1 mutation in adult human ventricular CMs. This table follows the format of Table 3.

## Discussion

There is an unmet need for developing new anti-arrhythmic drugs (see, e.g., [6, 10, 54]) for a whole series of cardiac related conditions. The scientific and regulatory path required for approval of a new compound are, however, both long and extremely costly [55, 56]. These challenges motivate the search for alternatives, and one plausible approach is to search for combinations of existing drugs. Although this sounds like a simple, and straightforward concept to test in a laboratory, the combination of a large group of different drugs applying a range of different drug concentrations quickly becomes a challenging endeavour. In addition, even if such lab experiments were conducted, the end result would be the right ‘mixed’ compound for the animal cells or hiPSC-CMs under consideration, and not actually a combination therapy suited for adult humans. Using mathematical models, this changes. In principle we are in position to use mathematical models to identify a precise mixed compound for normalizing the AP waveform and thus stabilizing adult human CMs. Since we can also compute the ideal compounds for hiPSC-CMs and rabbit CMs, the suggested combination therapy can be tested in order to gain insight into its applicability.

Our main aim in this study is to use mathematical models of the effects of well characterized existing drugs to find optimal combinations of these drugs that repair the effect of a given mutation. Specifically, we need information about how the drugs affect the ion currents governing the AP waveform and Ca^2+^ transients of the wild type and mutant cells. Here, we have provided an example of how a small collection of known drugs can be combined to ‘define’ a mixed compound that, in simulations, almost completely repairs the AP properties of the mutant myocytes. Our results are based on measured properties of the drugs under consideration, but our computational endpoints are purely theoretical in the sense that the resulting combination therapy has not been tested in the lab. However, the results are specified in a way that enables laboratory testing. In this section, we will summarize the method, point to possible applications and discuss limitations and possible weaknesses.

### Pharmaceutical considerations

As shown in Table 1 and Fig 4, one of the key insights from our computational approach for ‘correcting’ the dramatically shortened APD and depressed intracellular Ca^2+^ transient that are both hallmark features of the SQT1 syndrome is that a combination of two different approved cardiac drugs can be very effective. The electrophysiological principles that underpin this finding are worth reviewing. Veratridine, one of the compounds or drugs, that we have identified as being essential for restoring the APD waveform acts mainly by increasing the amplitude of one particular transmembrane current: the slowly inactivating or late Na^+^ current, *I*_NaL_ [57]. This small inward current can produce very significant changes in the plateau of the action potential for a number of different reasons. First, although *I*_NaL_ is small the membrane resistance at the plateau of the AP is relatively high (approximately three times larger than at the resting membrane potential). Second, *I*_NaL_ shows very little voltage-dependent inactivation and therefore provides an almost constant depolarizing influence over a broad range of membrane potentials [58]. In essence, therefore, *I*_NaL_ functions very similarly to the effects of the relatively long applied stimulus currents that were used by Wood, Heppner and Weidmann [59] in their original classical demonstration of the effects of plateau height and duration of the action potential waveform on ventricular contractility.

Our quite broadly based survey and related analyses of approved drugs that may be effective in restoring the dramatically shortened action potential which is characteristic of the SQT1 syndrome, and is caused by a mutation-induced, very marked enhancement of the K^+^ current, *I*_Kr_, has identified disopyramide as an effective antidote; and a potent component of a drug combination that can restore the ventricular AP waveform. Once again, this finding has an established functional basis. Perhaps the primary reason for the effectiveness of disopyramide (at the concentrations identified as being effective by our computational analysis) is that this drug potently blocks *I*_Kr_ (Table 1). In addition, since the initiation of repolarization of the mammalian ventricular action potential is known to be regenerative (that is it exhibits all-or-none behaviour), the dynamics of the size of *I*_Kr_ as well as its average amplitude are critical for initiating the final repolarization phase of the action potential (cf. [60]). Specifically, the blocking actions of disopyramide can significantly reduce the transient increase in the outward component of *I*_Kr_ that is produced during the final phase of repolarization due to the intrinsic inwardly rectifying property of this particular time- and voltage-dependent K^+^ conductance. In summary, interacting effects of enhancement of *I*_NaL_ and separate synergistic block of *I*_Kr_ can dramatically lengthen APD and restore the intracellular Ca^2+^ transient to very near its control or baseline contour [61]. It is also well known that even small changes in the rates of repolarization of the action potential can significantly alter the intracellular Ca^2+^ transient and associated ventricular contractility [62, 63].

### Method for finding optimal, combined compounds

We have used the method outlined above to find candidate combinations of known drugs that are effective in repairing the effect of the SQT1 mutation in three cases: hiPSC-CMs, rabbit CMs and adult human CMs. Note, however, that the procedure introduced here can similarly be applied to other mutations. Suppose a mutation changes the dynamics of one (or several) currents. The aim is then to find an optimal combination of a collection of *K* existing drugs that can ‘repair’ the effect of the mutation. The information needed to apply the method described above is how all *K* drugs affect the ion currents of the mutant myocytes. Here, we have used simple IC_50_/EC_50_ models to represent the effect of the drugs on the individual ion currents. In addition, an accurate AP model of each type of mutant myocyte is needed. With this information, we can run simulations to identify an optimal drug that can repair the AP of the mutant myocyte, as judged by alignment with the quantitative biomarkers for the AP waveform and the Ca^2+^ transient of a wild type myocyte. A feature of our method is that both the set of known drugs, the set of biomarkers, and the model of the effect of the drug, can readily be changed to address additional goals.

### Extension to other mutations: The requirement for adequate data sets

In this paper, we have focused on applying the computational method for finding optimal drug combinations to myocytes affected by one type of SQT1 mutation, N588K. The main reason for this is the availability of data on how drugs affect the mutated *I*_Kr_ current; see [28–30]. Our method also requires information on how the drugs affect all the ion currents of the mutant myocyte that is unaffected by the mutation, but this type of data is more generally available; see, e.g., [45, 46, 64–68]. In order to apply the computational method to identify drug combinations capable of repairing the effects of other mutations, information concerning how different drugs alter channels targeted by the mutation would preferably have to be obtained. Otherwise, only characterized drugs that act on channels not affected by the mutation could be included in the search. In the best case, we would apply data directly describing the effects of drugs on mutated channels collected from voltage-clamp measurements, particularly with full dose-response relationships (as in, e.g., [30]). An alternative is to generate hiPSC-CMs from a patient with the mutation (as in, e.g., [28, 29]) and use a computational procedure to indirectly predict the effect of the drugs on individual channels (including those affected by the mutation) based on measurements of the hiPSC-CM AP for different drug doses (see, e.g., [12, 32]). In future work, assuming access to data on how a collection of drugs of interest act on other mutations, it would be possible to repeat the steps we have taken here to devise optimal, theoretical, drugs for repairing the AP properties of the mutant cells.

### The optimal drug combination: Few drugs with high doses or many drugs with low doses?

As shown in Fig 3 and illustrated in Fig 4, our analysis reveals that the combination of two drugs can almost completely repair the effect of the SQT1 mutation on the AP waveform. However, this comes with a ‘price’ of needing relatively high doses, which are generally not clinically applicable due to off-target interactions and their resulting side effects. From Fig 6 we see that the doses can be significantly reduced if we include several (more than two) drugs in the combination. In fact, by requiring that all drugs have a concentration below 50% of their lowest EC_50_, we can completely repair the effect of the mutation using a combination of five drugs; see Fig 7.

### Modeling the effect of a drug

Modeling the effects of various drugs in the setting of cardiac arrhythmia has received considerable attention and a general introduction is provided in [69]. The two most common approaches to modeling the effect of drugs on ion currents of CMs are based on Markov models and IC_50_-models. Markov models (see, e.g., [21, 27, 70–72]) are much more detailed and, at least in some cases, closely tied to the molecular composition and biophysical properties of the channel. The disadvantage is that these models require very detailed data sets on every individual current and this is often not available. Ideally, in order to parameterize a Markov model properly, data from single channel measurements should be used (see, e.g., [73–76]), and such data are not commonly available. In contrast, the IC_50_-type modeling that we have used (see, e.g., [77–79]) is more straightforward and can be estimated based on few biomarkers (see, e.g., [33]). In the S1 Text, we give an example where we compare an IC_50_-model with a comprehensive Markov model from [80]. We have used this specific case because the Markov model from [80] is completely specified with all necessary parameters. In this Supplementary section, we show that these two ion channel model alternatives give similar results. Nevertheless, we would have preferred to use accurate models based on Markov models, but the necessary data is not presently available. If such data become available in the future, the approach described in the present paper could straightforwardly be extended to represent drug effects in terms of changes in specified states in Markov models instead of by the simple IC_50_/EC_50_ models used in this report.

As mentioned above, we assume that the effect of two drugs can be approximated by multiplying the individual effects of the two drugs. The justification of this approximation is as follows: Suppose the open probability of a certain ion channel is given by *o*. If we apply a blocker referred to as *A* to this channel, we assume that a certain fraction, *μ*_*A*_ ≤ 1, of the channels will be blocked (see (7)). After the application of this drug, the open probability of the channel is *μ*_*A*_*o*. Next, we assume that we have another drug, denoted by *B*. This drug is also a blocker and it blocks a fraction *μ*_*B*_ ≤ 1 of the open channels. By first applying the drug *A*, the open probability is *μ*_*A*_*o*, and then, by applying the drug *B* the open probability becomes *μ*_*B*_*μ*_*A*_*o*. Thus, we have assumed that the binding of the two blockers is non-interactive (strictly independent), i.e., they neither compete nor allosterically facilitate each others binding. In the S1 Text, we further discuss the question concerning the multiplicative effect of drug compounds. Using recent measurements from [81] we indicate that the effect of two blockers can be approximated by multiplying the effect of the two drugs. We have been unable to find more data on combined drug effects and therefore the assumption that we can multiply the effect remains *an assumption* that needs further consideration in future work.

It should also be noted the IC_50_-values reported in the literature can vary significantly. In [82, 83] it is argued that the reason for these differences may be the lack of uniformly accepted comprehensive protocols for measuring IC_50_-values.

### The assumption of functional invariance of ion channels

The approach for identifying optimal drug combinations for repairing the effect of mutations described in this paper relies on using pre-identified characteristics of how a number of drugs affect individual ion channels. We have applied the assumption that the function of an individual ion channel is the same for different species and for different levels of maturity. Based on this, we can use information about how drugs affect ion channels in expression systems or in hiPSC-CMs [28–30, 45, 46], and assume that the drug effect on the *individual channels* would be the same for adult human or rabbit cells, even though the drug effects on the action potential waveform may be different because of differences in the density of the various ion channels in the respective membranes.

The assumption of functional invariance of ion channels has previously been used to translate drug effects on the action potential waveform from one species to another and between hiPSC-CMs and adult ventricular CMs [12, 31–33]. This type of mapping of AP biomarkers is also possible without using the assumption that the function of an ion channel is identical for different species and maturity levels by using a regression-based translation approach [84]. This was, to our knowledge, the first proposed computational approach for translating between species or between hiPSC-CMs and adult human CMs, and relies on constructing a regression model by relating features of the source type of cell (e.g., hiPSC-CMs) to features of the target type of cell (e.g., adult ventricular CMs) under the same conditions.

### Variation of ion channel densities between individuals

In our computations, we consider models of hiPSC-CMs, rabbit ventricular CMs and adult human ventricular CMs representing the dynamics underlying typical wild type and SQT1 APs and Ca^2+^ transients. However, in a population of individuals, the density of different types of ion channels is expected to vary between individuals [85–87], and the representative models considered here might not be sufficient to represent the dynamics underlying the APs and Ca^2+^ transients of each of these individuals. More specifically, the optimal drug doses found for an individual represented by the default model might not be suitable for an individual with a different density of ion channels. In the S1 Text, we investigate how well the optimal drug doses found for the default adult human model are able to repair the APs and Ca^2+^ transients in a few cases with perturbed ion channel densities. The results indicate that the optimal drug doses are able to repair the AP and Ca^2+^ transient of the perturbed models reasonably well, but not as well as for the default model considered in the optimization method. Thus, a potential extension of the computational procedure outlined in this paper could be to consider multiple variations of ion channel densities in the optimization procedure and search for optimal combinations of drugs that are robust with respect to variations in these ion channel densities.

### Previous attempts to utilize anti-arrhythmic drug combinations

The concept of combining two drugs in clinical cardiac electrophysiology in order to achieve advantageous (anti-arrhythmic) outcomes has been evaluated in both animal studies and clinical settings; see, e.g., [88–90]. However, this approach seems to have received relatively little attention during the past 15 years. The earlier papers on combined drug actions usually express the effect in terms of clinical characteristics that are difficult to use in order to evaluate our hypothesis of multiplicative blocks (see (9)). In [89] the APDs are measured, but the drugs applied are chosen in order to modulate different targets. Therefore, these results cannot be used in order to understand multiplicativity of drug effects in terms of block of only one channel.

## Conclusion

We have used computational methods to identify drug combinations that can ‘repair’ the effects of a mutation in mammalian ventricular myocytes. This method is based on information of how a collection of drugs affects the relevant ion channels. For the SQT1 mutation N588K and the resulting increase of the *I*_Kr_ current, we were able to identify a theoretical combination therapy that completely repairs the effect of this mutation as judged by a set of biomarkers. If relatively high drug doses can be utilized, the effect of the mutation can be repaired using only two drugs. If low doses are required, more individual drugs need to be applied in order to completely repair the effect of the mutation.

## Supporting information

S1 Text Supplementary information regarding the methods, results and discussion. (PDF)

S1 Data Underlying numerical data for figures. Excel spreadsheet containing the underlying numerical data of Figs 2, 4, 5, 6 and 7.
(XLSX)
