# Supplementary material for: A computational method for identifying an optimal combination of existing drugs to repair the action potentials of SQT1 ventricular myocytes
Source: PLoS Comput Biol. 2021 Aug 12;17(8):e1009233. doi: 10.1371/journal.pcbi.1009233 (PMC8360568; doi:10.1371/journal.pcbi.1009233)
Supplement: S1 Text — (PDF) [file pcbi.1009233.s001.pdf]

# S1 Text: Supplementary information

## S1.1 Base model specifications

We use the base model formulation from [1] to represent the AP and $Ca^{2+}$ transient of wild type and SQT1 ventricular cardiomyocytes. The full base model formulation and the specific hiPSC-CM and adult human ventricular CM parameterizations of the model are specified in [1]. In this study, we also make use of a rabbit ventricular myocyte version of the model. The parameters of the rabbit model are as specified for the adult human version of the model in [1], except for the parameter values specified in Table S1.

| Parameter | Value | Parameter | Value |
|---|---|---|---|
| $g_{\mathrm{Na}}$ | 3.78 mS/$\mu$F | $g_{\mathrm{to}}$ | 0.189 mS/$\mu$F |
| $g_{\mathrm{Kr}}$ | 0.048 mS/$\mu$F | $g_{\mathrm{Ks}}$ | 0.06 mS/$\mu$F |
| $g_{\mathrm{K1}}$ | 0.925 mS/$\mu$F | $g_{\mathrm{bCl}}$ | 0.0163 mS/$\mu$F |
| $\bar{I}_{\mathrm{NaK}}$ | 0.864 $\mu$A/$\mu$F | $\bar{I}_{\mathrm{NaCa}}$ | 17.64 $\mu$A/$\mu$F |
| $g_{\mathrm{CaL}}$ | 0.27 nL/($\mu$F ms) | $g_{\mathrm{bCa}}$ | 0.000132 mS/$\mu$F |
| $\bar{I}_{\mathrm{pCa}}$ | 0.068 $\mu$A/$\mu$F | | |

Table S1: Parameter values of the rabbit version of the base model; the remaining parameter values are the same as for the adult human ventricular model in [1].

### S1.1.1 Pacing protocol

For the hiPSC-CM, adult human and rabbit versions of the base model we use 0.2 Hz, 1 Hz and 2 Hz pacing frequencies, respectively. In the EMI model simulations (see Section S1.4) and in the simulations investigating the effect of varying the maximum conductances (see Section S1.8), we run an ODE simulation of 500 pacing cycles to update the initial conditions for each parameter change before starting the simulation. In the inversion procedure, we run a simulation for 50 or 100 pacing cycles for each of 20 or 5 iterations of the continuation method, and update the initial conditions in each iteration (see [1] and Section S1.3).

## S1.2 Software

Matlab code for the base model is found in the Supplementary Material of [1]. A finite difference code of the EMI model written in Matlab is available

at [2], and a finite element version of the EMI model is available at [3]. All optimizations in the present paper are performed using Matlab with the method described in detail in [1].

## S1.3 Technical specifications of the optimization method

In order to find the drug doses that minimize the cost function, we use the continuation-based optimization method applied in [1, 4, 5]. See, e.g., [1] for a detailed description of this method.

In the search for optimal doses of one or two drugs, we restrict all drug doses so that the maximal effect of the drug is at most 95% of its maximal possible effect, $E$. We use 20 continuation iterations with 96 randomly chosen combinations of doses in each iteration and run 15 Nelder-Mead iterations for each randomly chosen combination.

In the search for optimal combinations with stricter restrictions on the doses, we gradually increase the number of considered drugs as described in the 'Minimization procedure' section in the paper. In each application of the continuation algorithm in this procedure, we use 5 continuation iterations with 96 randomly chosen combinations of doses in each iteration and run 15 Nelder-Mead iterations for each randomly chosen combination.

## S1.4 Technical specification of the EMI model simulations

| Parameter | Value | Parameter | Value |
|---|---|---|---|
| $C_m$ | 1 $\mu$F/cm$^2$ | $R_g$ | 0.0015 k$\Omega$cm$^2$ |
| $C_g$ | 0.5 $\mu$F/cm$^2$ | $\Delta t$ | 0.005 ms |
| $\sigma_i$ | 4 mS/cm | $\Delta t_{\text{ODE}}$ | 0.001 ms |
| $\sigma_e$ | 20 mS/cm | $M_{\text{it}}$, $N_{\text{it}}$ | 1 |

Table S2: Parameter values of the EMI model simulations, see, e.g., [6].

### S1.4.1 Parameter values and domain geometry

The parameters applied in the EMI model simulations (see, e.g., [6]) are specified in Table S2. The geometry of a single ventricular myocyte is illustrated in Figure S1A. In the adult case, each myocyte is 150 $\mu$m long and has a radius varying from 8 $\mu$m to 11 $\mu$m. In the hiPSC-CM case, each myocyte

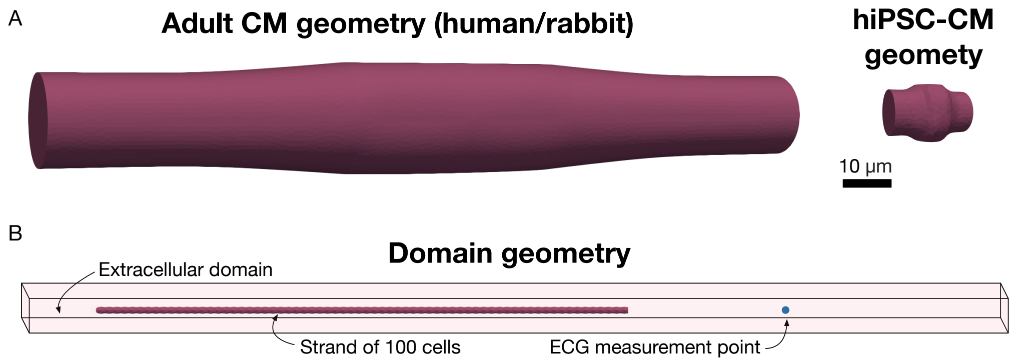


Figure S1: Illustration of the assumed geometry used in the EMI model simulations. A) The geometry of a single ventricular myocyte in the (i) adult CM and (ii) hiPSC-CM cases. We have used the same geometry for adult human and rabbit ventricular myocytes. B) Illustration of the full EMI model domain, consisting of a strand of 100 connected myocytes and a surrounding extracellular space. The ECG is measured at a point to the right of the cell strand. Note that the dimensions of the illustration in Panel B are not in scale; and that the scale bar only applies to panel A.

is 15 $\mu$m long and has a radius varying from 4 $\mu$m to 6 $\mu$m. In addition, we increase the gap junction resistance, $R_g$, in the hiPSC-CM case, to represent a weaker gap junction coupling between hiPSC-CMs as compared to adult CMs (see, e.g., [7, 8]). More specifically, $R_g$ is increased by a factor of 4, resulting in a default conduction velocity of approximately 5 cm/s, similar to what was observed in sheets of hiPSC-CMs in [9, 10, 11].

The full EMI model domain is illustrated in Figure S1B. We consider a strand of 100 connected myocytes surrounded by an extracellular domain. We initiate a propagating AP through these myocytes by setting the intracellular potential of the first two (leftmost) myocytes to $-10$ mV. We apply a homogeneous Dirichlet boundary condition at the extracellular left boundary and homogeneous Neumann boundary conditions on the remaining extracellular boundaries. The minimum distance from the extracellular boundary to the myocyte membrane in the $y$- and $z$-directions is 5 $\mu$m in the adult case and 10 $\mu$m in the hiPSC-CM case. The distance from the extracellular left boundary to the first myocyte is 0.5 cm and the distance from the extracellular right boundary to the last myocyte is 1.5 cm.

Table S3 provides information on the size of the computational problems associated with the EMI model simulations.

## Computational complexity

| | hiPSC-CMs | | | Rabbit CMs | | | Adult human CMs | | |
|---|---|---|---|---|---|---|---|---|---|
| | $N_{\mathrm{E}}$ | $N_{\mathrm{M}}$ | $N_{\mathrm{I}}$ | $N_{\mathrm{E}}$ | $N_{\mathrm{M}}$ | $N_{\mathrm{I}}$ | $N_{\mathrm{E}}$ | $N_{\mathrm{M}}$ | $N_{\mathrm{I}}$ |
| Number of nodes | 9,535 | 7,016 | 7,754 | 13,032 | 6,616 | 7,198 | 13,032 | 6,616 | 7,198 |
| Number of state variables for M, $N_{\mathrm{S}}$ | 25 | | | 25 | | | 25 | | |
| Simulation time (ms) | 500 | | | 250 | | | 350 | | |
| | $\Delta t_{\mathrm{PDE}}$ | $\Delta t_{\mathrm{ODE}}$ | | $\Delta t_{\mathrm{PDE}}$ | $\Delta t_{\mathrm{ODE}}$ | | $\Delta t_{\mathrm{PDE}}$ | $\Delta t_{\mathrm{ODE}}$ | |
| Time step (ms) | 0.005 | 0.001 | | 0.005 | 0.001 | | 0.005 | 0.001 | |
| | $N_{\mathrm{PDE}}$ | $N_{\mathrm{ODE}}$ | | $N_{\mathrm{PDE}}$ | $N_{\mathrm{ODE}}$ | | $N_{\mathrm{PDE}}$ | $N_{\mathrm{ODE}}$ | |
| Number of time points | 100,000 | 500,000 | | 50,000 | 250,000 | | 70,000 | 350,000 | |
| Total number of computed values, $(N_{\mathrm{E}} + N_{\mathrm{I}})N_{\mathrm{PDE}}$ $+N_{\mathrm{M}}N_{\mathrm{S}}N_{\mathrm{ODE}}$ | $8.9 \cdot 10^{10}$ | | | $4.2 \cdot 10^{10}$ | | | $5.9 \cdot 10^{10}$ | | |

Table S3: Illustration of the sizes of the computational requirements associated with the EMI model simulations. The top row reports the number of finite element nodes in the extracellular (E), membrane (M) and intracellular (I) domains. The second row reports the number of state variables of the membrane model. The third row reports the simulation times used in the simulations of hiPSC-CMs, rabbit CMs and adult human CMs, respectively. The lower two rows report the time step used for the ordinary differential equation (ODE) part of the simulation (for the state variables of the membrane) and the partial differential equation (PDE) part of the simulation, and the associated number of time steps for the full simulation time. Finally, the bottom row reports the total number of solution values computed during these simulations.

### S1.4.2 Definition of the pseudo-ECG and QT interval

The pseudo-ECG is measured at a point 1 cm to the right of the last myocyte (only for the adult human and rabbit cases). In order to set up a pseudo-ECG in these cases, we introduce transmural heterogeneity along the myocyte strand. More specifically, we let the first 25 myocytes define an endocardial region, the next 35 myocytes define a midmyocardial region and the last 40 myocytes define an epicardial region. The default base model parameters define the parameters of the epicardial region. In the endocardial region, the $I_{\mathrm{Ks}}$ and $I_{\mathrm{to}}$ current densities are reduced to 31% and 1%, respectively, compared to the epicardial region. In the midmiocardial region, these currents are reduced to 11% and 85%, respectively [12].

The QT interval is computed as the time from the start of the QRS-complex of the computed pseudo-ECG to the end of the T-wave. More specifically, we extract the first and last time points of the simulation when the absolute value of the extracellular potential in the measurement point is above 1% of the maximum absolute value achieved at the measurement point.

### S1.4.3 Definition of conduction velocity

The conduction velocity is computed as the distance between the center of myocyte number 70 and myocyte number 20 divided by the duration of the time interval between the membrane potential in the center of the two myocytes reaches a value above 0 mV.

## S1.5 Supplementary inversion results

In this section, some supplementary results from the computational procedure are reported. In Figures S2 and S3, the AP and $Ca^{2+}$ transients of the SQT1 models following the application of optimal doses of a single drug applied in an attempt to repair the SQT1 mutation in hiPSC-CMs and rabbit CMs are plotted and compared to the wild type and SQT1 AP and $Ca^{2+}$ transients with no drug applied. Tables S4 and S5 report the values of a number of biomarkers from the wild type and SQT1 models, in addition to the SQT1 cases after optimal drug doses are applied. Tables S6 and S7 report the optimal doses found in each case.

In addition, Tables S8 and S9 report biomarkers for the SQT1 models for hiPSC-CMs and rabbit ventricular CMs with the optimal combination of five drugs with the restriction $D \leq \min(\mathrm{EC}_{50})/2$ applied, and Tables S10 and S11 report the optimal drug doses in these optimal drug combinations.

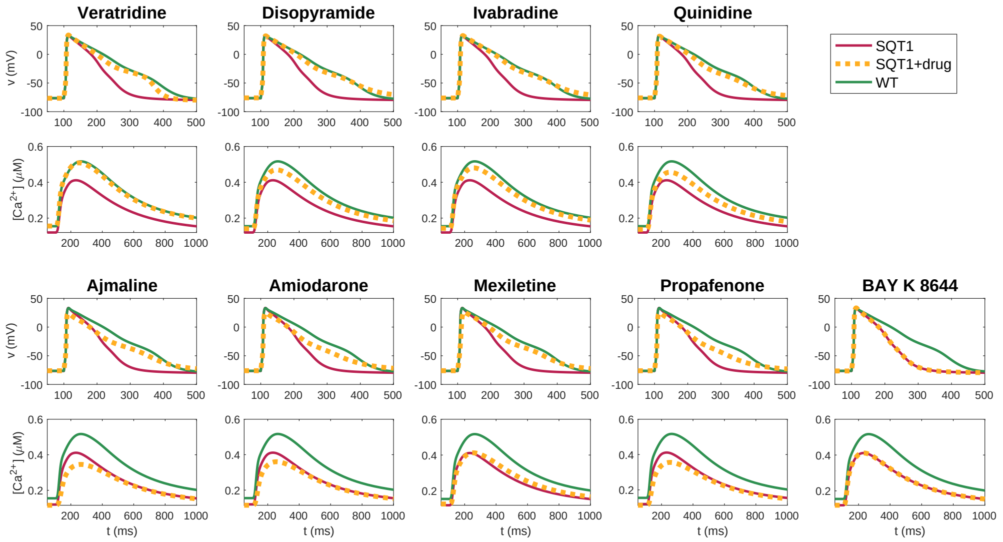


Figure S2: AP and $Ca^{2+}$ transients for hiPSC-CMs in the wild type case, in the SQT1 case, and in the SQT1 case following application of an optimal dose of each of the drugs listed in Table 1 in the paper.

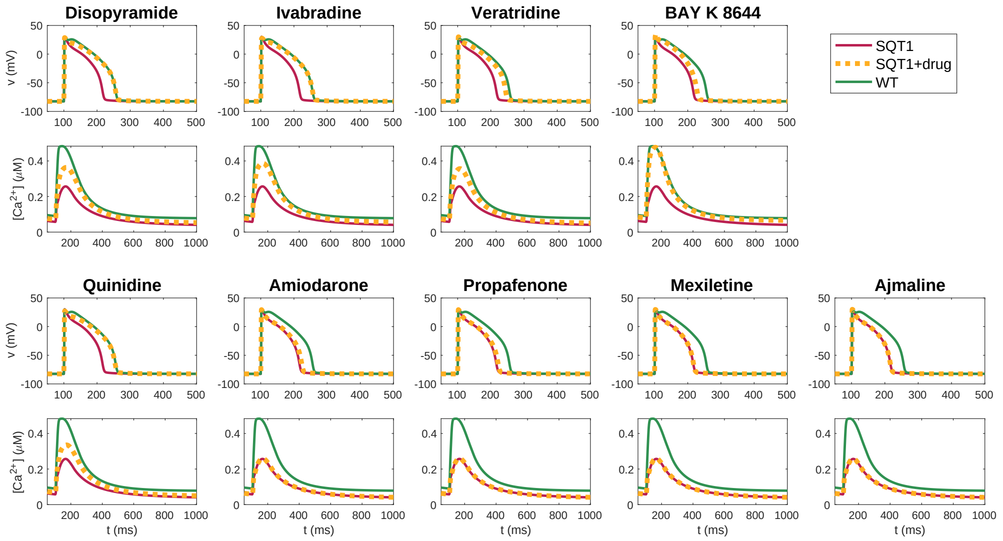


Figure S3: AP and $Ca^{2+}$ transients for rabbit ventricular CMs in the wild type case, in the SQT1 case, and in the SQT1 case after application of the optimal dose of each of the drugs of Table 1 in the paper.

| | Cost | APD50 | | APD90 | | $dvdt_{max}$ | | CV | |
|---|---|---|---|---|---|---|---|---|---|
| | function | ms | % from WT | ms | % from WT | mV/ms | % from WT | cm/s | % from WT |
| WT (no drug) | 0 | 177 | | 325 | | 31 | | 5 | |
| SQT1 (no drug) | 8.1 | 106 | −40% | 188 | −42% | 32 | +3% | 5 | +1% |
| Combination drug | 0.6 | 160 | −10% | 325 | −0% | 31 | −0% | 5 | −2% |
| Veratridine | 1.8 | 151 | −15% | 288 | −12% | 32 | +4% | 5 | −1% |
| Disopyramide | 2.1 | 153 | −13% | 348 | +7% | 26 | −14% | 4 | −8% |
| Ivabradine | 2.8 | 156 | −12% | 346 | +6% | 21 | −31% | 4 | −17% |
| Quinidine | 2.8 | 148 | −16% | 329 | +1% | 24 | −22% | 4 | −11% |
| Ajmaline | 3.8 | 140 | −21% | 325 | −0% | 26 | −14% | 4 | −8% |
| Amiodarone | 5.0 | 131 | −26% | 319 | −2% | 21 | −30% | 4 | −16% |
| Mexiletine | 5.5 | 145 | −18% | 325 | −0% | 10 | −66% | 3 | −41% |
| Propafenone | 5.7 | 127 | −28% | 302 | −7% | 21 | −31% | 4 | −16% |
| BAY K 8644 | 5.8 | 106 | −40% | 188 | −42% | 32 | +3% | 5 | +1% |

Table S4: Cost function and biomarker values of the hiPSC-CM models for wild type and SQT1 with no drugs present, as well as for the SQT1 model with the optimal combination of two drugs or after application of the optimal dose of the individual drugs. We report the cost function value, the action potential durations, APD50 and APD90, the maximal upstroke velocity of the action potential, $dvdt_{max}$, and the conduction velocity, CV. In the SQT1 cases, we also report the percent difference from the wild type case.

| | Cost | APD50 | | APD90 | | $dvdt_{max}$ | | CV | | QT | |
|---|---|---|---|---|---|---|---|---|---|---|---|
| | function | ms | % from WT | ms | % from WT | mV/ms | % from WT | cm/s | % from WT | ms | % from WT |
| WT (no drug) | 0 | 133 | | 157 | | 156 | | 45 | | 163 | |
| SQT1 (no drug) | 7.3 | 94 | −29% | 117 | −25% | 157 | +0% | 45 | +0% | 132 | −19% |
| Combination drug | 0.4 | 135 | +1% | 160 | +2% | 153 | −2% | 44 | −2% | 153 | −6% |
| Disopyramide | 2.3 | 132 | −0% | 157 | +0% | 140 | −11% | 43 | −5% | 166 | +2% |
| Ivabradine | 2.6 | 133 | +0% | 157 | +0% | 114 | −27% | 39 | −14% | 164 | +1% |
| Veratridine | 3.0 | 121 | −9% | 145 | −7% | 156 | +0% | 45 | −1% | 142 | −13% |
| BAY K 8644 | 3.0 | 106 | −20% | 129 | −18% | 156 | +0% | 45 | −1% | 121 | −26% |
| Quinidine | 3.4 | 132 | −1% | 156 | −1% | 119 | −24% | 40 | −12% | 168 | +3% |
| Amiodarone | 6.7 | 101 | −24% | 124 | −21% | 151 | −3% | 44 | −1% | 139 | −14% |
| Propafenone | 6.9 | 99 | −25% | 123 | −22% | 151 | −3% | 44 | −2% | 138 | −15% |
| Mexiletine | 7.3 | 95 | −28% | 118 | −25% | 153 | −2% | 45 | −1% | 133 | −18% |
| Ajmaline | 7.3 | 94 | −29% | 117 | −25% | 157 | +0% | 45 | +0% | 132 | −19% |

Table S5: Cost function and biomarker values of the rabbit ventricular CM models for wild type and SQT1 with no drugs present, as well as for the SQT1 model with the optimal combination of two drugs or the optimal dose of the individual drugs applied. We report the cost function value, the action potential durations, APD50 and APD90, the maximal upstroke velocity of the action potential, $dvdt_{max}$, the conduction velocity, CV, and the QT interval. In the SQT1 cases, we also report the percent difference from the wild type case.

| Drug | Optimal dose | (max % of $E$) | | % change of currents $I_{Kr}$ | $I_{CaL}$ | $I_{Na}$ | $I_{NaL}$ | $I_f$ |
|---|---|---|---|---|---|---|---|---|
| Combination of two drugs | 0.502 $\mu$M<br>8.63 $\mu$M | (58%)<br>(41%) | veratridine<br>disopyramide | −41.1% | −0.6% | −1.2% | +104.7% | +0.0% |
| Veratridine | 1.86 $\mu$M | (95%) | | +0.0% | +0.0% | +0.0% | +171.0% | +0.0% |
| Disopyramide | 131 $\mu$M | (78%) | | −78.1% | −8.7% | −13.4% | +0.0% | +0.0% |
| Ivabradine | 42.6 $\mu$M | (77%) | | −77.2% | +0.0% | −33.0% | +0.0% | −50.3% |
| Quinidine | 22.1 $\mu$M | (73%) | | −73.1% | −12.6% | −22.1% | +0.0% | +0.0% |
| Ajmaline | 73 $\mu$M | (61%) | | −51.2% | −61.0% | −14.4% | +0.0% | +0.0% |
| Amiodarone | 1.41 $\mu$M | (68%) | | −67.8% | −51.5% | −30.5% | −31.9% | +0.0% |
| Mexiletine | 444 $\mu$M | (69%) | | −61.2% | −31.5% | −68.8% | +0.0% | +0.0% |
| Propafenone | 1.65 $\mu$M | (60%) | | −59.6% | −51.4% | −31.7% | −30.9% | +0.0% |
| BAY K 8644 | 0.000282 $\mu$M | (0.015%) | | +0.0% | +0.0% | +0.0% | +0.0% | +0.0% |

Table S6: Optimal doses and associated changes of currents identified for single drugs or a combination of two drugs selected for repairing the SQT1 mutation in hiPSC-CMs. This table has the same structure as Table 3 in the paper.

| Drug | Optimal dose | (max % of $E$) | | % change of currents $I_{Kr}$ | $I_{CaL}$ | $I_{Na}$ | $I_{NaL}$ | $I_f$ |
|---|---|---|---|---|---|---|---|---|
| Combination of two drugs | 1.86 $\mu$M<br>15.3 $\mu$M | (95%)<br>(50%) | veratridine<br>disopyramide | −49.6% | −1.1% | −2.1% | +171.0% | +0.0% |
| Disopyramide | 122 $\mu$M | (77%) | | −77.3% | −8.2% | −12.8% | +0.0% | +0.0% |
| Ivabradine | 38.5 $\mu$M | (75%) | | −75.3% | +0.0% | −30.9% | +0.0% | −47.8% |
| Veratridine | 1.86 $\mu$M | (95%) | | +0.0% | +0.0% | +0.0% | +171.0% | +0.0% |
| BAY K 8644 | 0.0642 $\mu$M | (60%) | | +0.0% | +108.9% | +0.0% | +0.0% | +0.0% |
| Quinidine | 29.7 $\mu$M | (78%) | | −78.5% | −16.2% | −27.6% | +0.0% | +0.0% |
| Amiodarone | 0.0492 $\mu$M | (28%) | | −28.2% | −12.4% | −4.0% | −10.9% | +0.0% |
| Propafenone | 0.131 $\mu$M | (20%) | | −20.0% | −9.7% | −4.5% | −4.4% | +0.0% |
| Mexiletine | 4.97 $\mu$M | (2.4%) | | −1.7% | −0.5% | −2.4% | +0.0% | +0.0% |
| Ajmaline | 8.88e-16 $\mu$M | (0%) | | +0.0% | +0.0% | +0.0% | +0.0% | +0.0% |

Table S7: Optimal doses and associated change of currents found for single drugs or a combination of two drugs selected for repairing the SQT1 mutation in rabbit ventricular CMs. This table has the same structure as Table 3 in the paper.

| | Cost function | APD50 ms | APD50 % from WT | APD90 ms | APD90 % from WT | $dvdt_{max}$ mV/ms | $dvdt_{max}$ % from WT | CV cm/s | CV % from WT |
|---|---|---|---|---|---|---|---|---|---|
| WT (no drug) | 0 | 177 | | 325 | | 31 | | 5 | |
| SQT1 (no drug) | 8.1 | 106 | −40% | 188 | −42% | 32 | +3% | 5 | +1% |
| Combination drug | 1.1 | 156 | −12% | 325 | −0% | 28 | −8% | 5 | −5% |

Table S8: Cost function value and biomarker values for the SQT1 hiPSC-CM model with the optimal combination of five drugs with the restriction $D \leq \min(EC_{50})/2$ applied. This table follows the format of Table S6.

|  | $C$ | APD50 |  | APD90 |  | $\text{dvdt}_{\text{max}}$ |  | CV |  | QT |  |
|---|---|---|---|---|---|---|---|---|---|---|---|
|  |  | ms | % from WT | ms | % from WT | mV/ms | % from WT | cm/s | % from WT | ms | % from WT |
| WT (no drug) | 0 | 133 |  | 157 |  | 156 |  | 45 |  | 163 |  |
| SQT1 (no drug) | 7.3 | 94 | −29% | 117 | −25% | 157 | +0% | 45 | +0% | 132 | −19% |
| Combination drug | 0.8 | 130 | −2% | 155 | −1% | 140 | −10% | 43 | −5% | 156 | −4% |

Table S9: Cost function value and biomarkers for the SQT1 rabbit ventricular model with the optimal combination of five drugs with the restriction $D \leq \min(\text{EC}_{50})/2$ applied. This table follows the format of Table S6.

| Drug | Optimal dose | (max % of $E$) |  | % change of currents $I_{\text{Kr}}$ | $I_{\text{CaL}}$ | $I_{\text{Na}}$ | $I_{\text{NaL}}$ | $I_{\text{f}}$ |
|---|---|---|---|---|---|---|---|---|
| Combination of five drugs | 7.63 $\mu$M | (39%) | disopyramide | −66.7% | +11.2% | −8.9% | +35.9% | −7.0% |
|  | 3.71 $\mu$M | (31%) | quinidine |  |  |  |  |  |
|  | 0.213 $\mu$M | (20%) | veratridine |  |  |  |  |  |
|  | 3.16 $\mu$M | (20%) | ivabradine |  |  |  |  |  |
|  | 0.012 $\mu$M | (8.1%) | BAY K 8644 |  |  |  |  |  |

Table S10: Optimal doses of a combination of five drugs with the restriction $D \leq \min(\text{EC}_{50})/2$ found for repairing the SQT1 mutation in hiPSC-CMs. This table follows the format of Table 3 in the paper.

| Drug | Optimal dose | (max % of $E$) |  | % change of currents $I_{\text{Kr}}$ | $I_{\text{CaL}}$ | $I_{\text{Na}}$ | $I_{\text{NaL}}$ | $I_{\text{f}}$ |
|---|---|---|---|---|---|---|---|---|
| Combination of five drugs | 7.85 $\mu$M | (40%) | disopyramide | −73.0% | +9.6% | −12.3% | +35.4% | −12.9% |
|  | 4.03 $\mu$M | (33%) | quinidine |  |  |  |  |  |
|  | 6.21 $\mu$M | (33%) | ivabradine |  |  |  |  |  |
|  | 0.211 $\mu$M | (20%) | veratridine |  |  |  |  |  |
|  | 0.0112 $\mu$M | (7.3%) | BAY K 8644 |  |  |  |  |  |

Table S11: Optimal doses of a combination of five drugs with the restriction $D \leq \min(\text{EC}_{50})/2$ found for repairing the SQT1 mutation in rabbit ventricular CMs. This table follows the format of Table 3 in the paper.

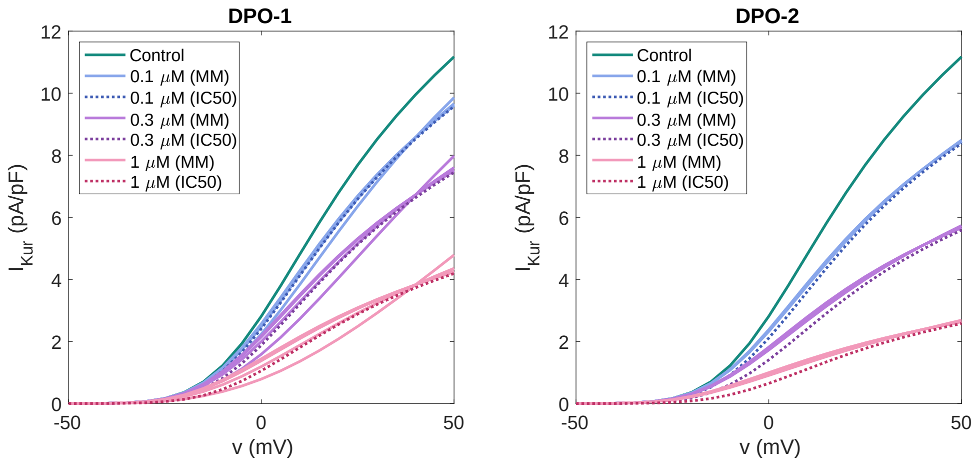


Figure S4: Steady state $I_{\mathrm{Kur}}$ in the control case and for three different doses of two drugs (DPO-1 and DPO-2) modeled using a Markov model from [15]. The solid lines with the same color represent different parameterizations of the drug effects from [15]. The dotted lines represent a simplified $\mathrm{IC}_{50}$-based modeling of the drug effects.

## S1.6 Comparison of $\mathrm{IC}_{50}$-based modeling of drug effects to Markov models of drug effects

A potential limitation of our computational procedure is that we use a very simple model for reconstructing the ion channel mediated effect of a drug (see (5) in the paper). Much more detailed models for drug effects have been published. Typically, these models are expressed in the form of Markov models (see, e.g., [13, 14]). An advantage of these more detailed models is that, in principle, they are able to yield a much more realistic and detailed representation of the actual effect of drugs, including, e.g., voltage and use–dependent drug effects. On the other hand, a disadvantage is that the Markov models typically introduce a large number of parameters that must be parameterized accurately. This requires information from detailed measurements of the effect of the drug on the ion channels.

In order to begin to assess of the difference between a Markov model for drug effects and the more simplified $\mathrm{IC}_{50}$-based modeling used in our study, we consider a Markov model for the $I_{\mathrm{Kur}}$ current from [15]. Based on measurements of the effect of two drugs (DPO-1 and DPO-2) on this current, four different model parameterizations for the drugs were considered, see [15]. In Figure S4, we have plotted the steady-state $I_{\mathrm{Kur}}$ currents as a function of the membrane potential in the control case and for three different doses of the

drugs. The solid lines with the same color show the solution for each of the four Markov model parameterizations. In addition, the dotted lines show the currents computed using a simple $IC_{50}$-based scaling of the control current. We observe that the $IC_{50}$-based modeling of the drug effects is in relatively good agreement with the full Markov model of drug effects. Because of this and since detailed measurements of drug effects needed to set up realistic Markov model representations of drugs effects are not always available, we have chosen to use a simple $IC_{50}$-based modeling of drug effects in this study.

## S1.7 Is the effect of separate drugs multiplicative?

A central assumption in our method is that if two drugs affect the same ion channel, their combined effect can be identified by multiplication of the respective effects. Using this assumption, we are able to identify effective drug combinations. But is the assumption reasonable? In order to assess the assumption, we ideally would have liked to see experiments showing the effect of two drugs individually, and then the effect of the combination of the drugs. However, any such data sets are hard to find. A very recent paper [16] is useful: the effect of hydroxychloroquine (HCQ) and azithromycin (AZM) are analysed. In Figure S5 we show the data from [16] for the effect on the $I_{\mathrm{Kr}}$ current by applying HCQ (left), AZM (middle), and the combination of HCQ and AZM (right). In the right panel we also show the estimated effect obtained by assuming that the combined effect of two drugs are multiplicative. We note that the approximation obtained by multiplication is qualitatively acceptable but that this approach is not very accurate for low doses of HCQ. For high doses, this approximation seems to be applicable.

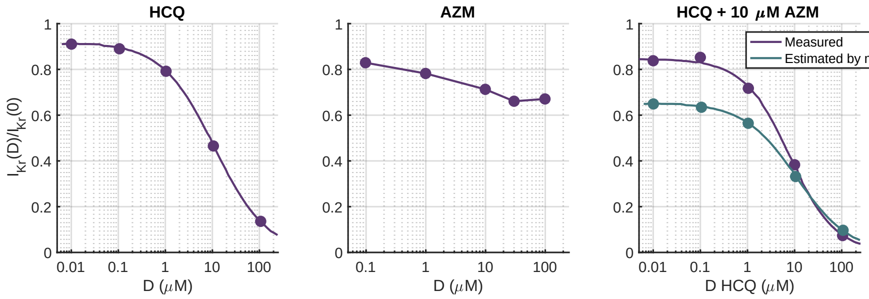


Figure S5: Data from S5 for the effect on the $I_{\mathrm{Kr}}$ current using varying doses of HCQ (left), AZM (middle) and the combination of HCQ and AZM (right). In the right panel, we also show the approximate effect of the combined drug obtained by multiplication of the individual drug effects.

## S1.8 Are the optimal drug doses also applicable for variations of ion channel densities?

In this subsection, we wish to investigate how effective the optimal drug doses identified for the default model of adult human ventricular CMs are if the ion channel densities are slightly different. More specifically, we multiply the maximum conductance of each of the channels by a factor between 0.75 and 1.25 and compare the APs and $Ca^{2+}$ transients for WT, SQT1 and SQT1 after application of the optimal combination of drugs. We consider five examples of randomly drawn perturbations of the maximum conductances, listed in Table S12, and for each case consider the drug doses identified to be optimal for the default version of the model. The upper panel of Figure S6 considers the optimal combination of two drugs, and the lower panel considers the optimal combination of five drugs with the restriction $D \leq \min(\text{EC}_{50})/2$. We observe that the drug combinations seem to be able to repair the SQT1 APs and $Ca^{2+}$ transients reasonably well for the perturbed models, but not quite as well as for the default model considered in the optimization procedure (see Figs 4 and 7 in the paper).

**Adjustment factors for the maximum conductances**

| Parameter | Case 1 | Case 2 | Case 3 | Case 4 | Case 5 |
|---|---|---|---|---|---|
| $g_{\text{Na}}$ | 0.8409 | 0.7798 | 1.2365 | 0.7802 | 0.9362 |
| $g_{\text{Kr}}$ | 0.8819 | 1.0910 | 1.0745 | 0.9496 | 0.8490 |
| $g_{\text{K1}}$ | 0.8227 | 0.7712 | 1.1502 | 1.0134 | 0.9949 |
| $g_{\text{NaK}}$ | 0.8180 | 0.7857 | 0.9769 | 0.9584 | 0.9198 |
| $g_{\text{CaL}}$ | 1.1846 | 1.0108 | 0.9662 | 1.0785 | 1.2258 |
| $g_{\text{pCa}}$ | 1.0398 | 0.7984 | 1.1626 | 1.0640 | 1.2102 |
| $g_{\text{to}}$ | 1.0250 | 1.1591 | 0.7917 | 0.8960 | 0.7763 |
| $g_{\text{Ks}}$ | 0.8225 | 1.1587 | 0.8166 | 0.9658 | 1.1189 |
| $g_{\text{bCl}}$ | 1.1765 | 1.1112 | 0.8367 | 0.7578 | 0.8845 |
| $g_{\text{NaCa}}$ | 1.0611 | 0.8250 | 0.9455 | 1.2420 | 0.9614 |
| $g_{\text{bCa}}$ | 0.9255 | 1.0798 | 1.1657 | 0.8336 | 1.0240 |
| $g_{\text{NaL}}$ | 1.0066 | 0.9856 | 1.1517 | 0.8031 | 1.2213 |

Table S12: Adjustment factors for the maximum conductances of the ion channels used in each of the perturbed parameterizations of the adult human ventricular base model in Figure S6.

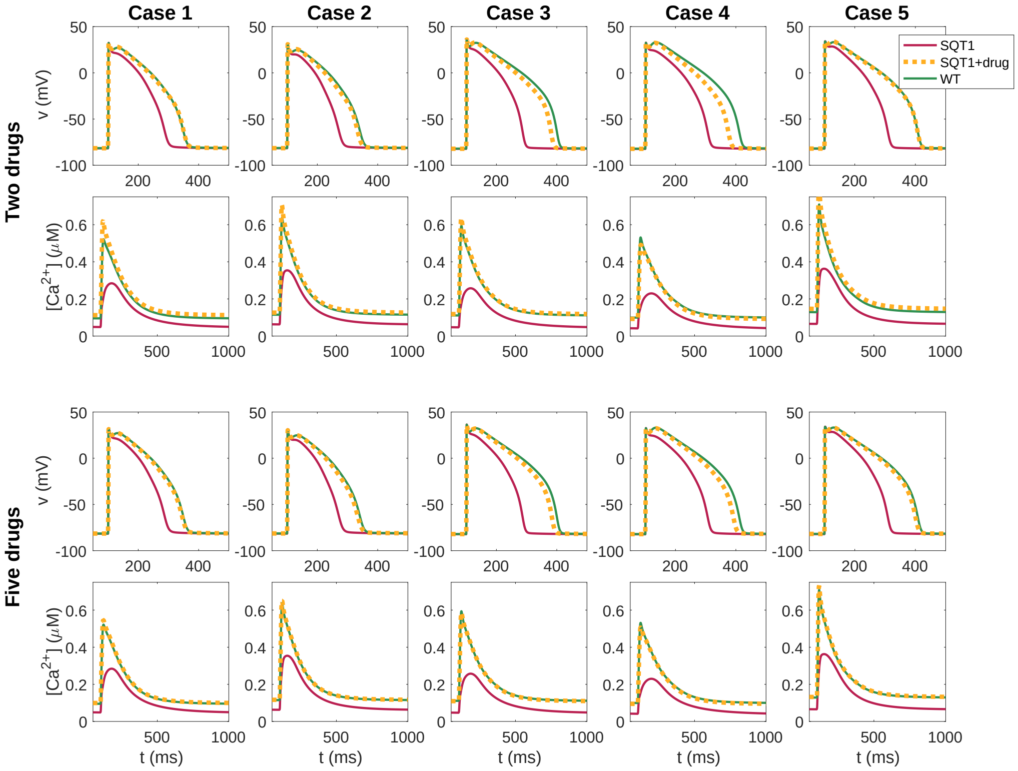


Figure S6: AP and $Ca^{2+}$ transients for adult human ventricular CMs in the wild type case (solid green), in the SQT1 case (solid red), and in the SQT1 case after the application of the optimal combination of two drugs (see Table 3 in the paper) or five drugs with the restriction $D \leq \min(\text{EC}_{50})/2$ (see Table 5 in the paper). For each column, the maximum conductances of the currents are adjusted by the adjustment factors specified in Table S12, but the drug doses are those identified for the default AP model parameterization.

# References


[1] Karoline Horgmo Jæger, Samuel Wall, and Aslak Tveito. Computational prediction of drug response in short QT syndrome type 1 based on measurements of compound effect in stem cell-derived cardiomyocytes. PLoS Computational Biology, 17(2):e1008089, 2021.

[2] Karoline Horgmo Jæger and Kristian Gregorius Hustad. Supplementary material (code) for the chapter "Operator splitting and finite difference schemes for solving the EMI model" appearing in "EMI: Cell-based Mathematical Model of Excitable Cells". https://doi.org/10.5281/zenodo.3707472, 2020.

[3] Miroslav Kuchta, Kent-Andre Mardal, and Marie Elisabeth Rognes. Software for EMI - Solving the EMI equations using finite element methods. https://doi.org/10.5281/zenodo.3769254, 2020.

[4] Karoline Horgmo Jæger, Verena Charwat, Bérénice Charrez, Henrik Finsberg, Mary M Maleckar, Samuel Wall, Kevin E Healy, and Aslak Tveito. Improved computational identification of drug response using optical measurements of human stem cell derived cardiomyocytes in microphysiological systems. Frontiers in Pharmacology, 10:1648, 2020.

[5] Karoline Horgmo Jæger, Sam Wall, Verena Charwat, Kevin Healy, and Aslak Tveito. Identifying drug response by combining measurements of the membrane potential, the cytosolic calcium concentration, and the extracellular potential in microphysiological systems. Frontiers in Pharmacology, 11:569489, 2021.

[6] Karoline Horgmo Jæger, Kristian Gregorius Hustad, Xing Cai, and Aslak Tveito. Efficient numerical solution of the EMI model representing the extracellular space (E), cell membrane (M) and intracellular space (I) of a collection of cardiac cells. Frontiers in Physics, 8:539, 2021.

[7] Matthew E Hartman, Dao-Fu Dai, and Michael A Laflamme. Human pluripotent stem cells: prospects and challenges as a source of cardiomyocytes for in vitro modeling and cell-based cardiac repair. Advanced Drug Delivery Reviews, 96:3–17, 2016.

[8] Nikki HL van den Heuvel, Toon AB van Veen, Bing Lim, and Malin KB Jonsson. Lessons from the heart: mirroring electrophysiological characteristics during cardiac development to in vitro differentiation of stem cell derived cardiomyocytes. Journal of Molecular and Cellular Cardiology, 67:12–25, 2014.

[9] Shin Kadota, Itsunari Minami, Nobuhiro Morone, John E Heuser, Konstantin Agladze, and Norio Nakatsuji. Development of a reentrant arrhythmia model in human pluripotent stem cell-derived cardiac cell sheets. European Heart Journal, 34(15):1147–1156, 2013.

[10] Masahide Kawatou, Hidetoshi Masumoto, Hiroyuki Fukushima, Gaku Morinaga, Ryuzo Sakata, Takashi Ashihara, and Jun K Yamashita. Modelling Torsade de Pointes arrhythmias in vitro in 3D human iPS cell-engineered heart tissue. Nature Communications, 8(1):1–11, 2017.

[11] Rami Shinnawi, Naim Shaheen, Irit Huber, Assad Shiti, Gil Arbel, Amira Gepstein, Nimer Ballan, Noga Setter, Anke J Tijsen, Martin Borggrefe, et al. Modeling reentry in the short QT syndrome with human-induced pluripotent stem cell–derived cardiac cell sheets. Journal of the American College of Cardiology, 73(18):2310–2324, 2019.

[12] Kazutaka Gima and Yoram Rudy. Ionic current basis of electrocardiographic waveforms: a model study. Circulation Research, 90(8):889–896, 2002.

[13] Colleen E Clancy, Zheng I Zhu, and Yoram Rudy. Pharmacogenetics and anti-arrhythmic drug therapy: a theoretical investigation. American Journal of Physiology-Heart and Circulatory Physiology, 292(1):H66–H75, 2007.

[14] Aslak Tveito and Glenn Terje Lines. Computing Characterizations of Drugs for Ion Channels and Receptors Using Markov Models. Springer-Verlag, Lecture Notes, vol. 111, 2016.

[15] Joachim Almquist, Mikael Wallman, Ingemar Jacobson, and Mats Jirstrand. Modeling the effect of Kv1. 5 block on the canine action potential. Biophysical Journal, 99(9):2726–2736, 2010.

[16] Gongxin Wang, Xiaohui Tian, Chieh-Ju Lu, Hannali Flores, Piotr Maj, Kevin Zhang, Yanhong Niu, Luxi Wang, Yimei Du, Xinying Ji, et al. Mechanistic insights into ventricular arrhythmogenesis of hydroxychloroquine and azithromycin for the treatment of covid-19. bioRxiv, 2020.
